# Synthesis and investigation of optical properties and enhancement photocatalytic activity of TiO_2_–SnO_2_ semiconductor for degradation of organic compounds

**DOI:** 10.1038/s41598-024-78755-y

**Published:** 2024-11-13

**Authors:** Wagih Sadik, Abdelghaffar M. El-Demerdash, Adel William Nashed, Amr Ahmed Mostafa, Elsayed Lamie

**Affiliations:** Materials Science Department, Institute of Graduate Studies & Research, Alexandria University, Alexandria, Egypt

**Keywords:** Optical properties, TiO_2_–SnO_2_ semiconductor, Wastewater treatment, UV irradiation, Organic compounds, Chemistry, Energy science and technology, Materials science, Nanoscience and technology

## Abstract

Industrial wastewater treatment using UV irradiation in combination with oxidants or catalysts (TiO_2_) has attracted attention as a promising substitute for conventional methods. Studying the preparation and characterization of TiO_2_–SnO_2_ nanocomposites with different ratios, as well as their use in the enhanced photocatalytic degradation of acid red 37 dye in aqueous solution under UV irradiation as a model pollutant, are the goals of the research. The crystalline structures of the prepared nanomaterials were confirmed by XRD and the surface morphology of the samples was studied by TEM. The elemental compositions of the catalysts were confirmed by EDAX. The optical properties of the powder samples were analyzed with UV–Vis spectroscopy and their band gaps were estimated. The photocatalytic degradation was investigated using several advanced oxidation techniques using a batch photoreactor. The TiO_2_–SnO_2_ (90:10) nanocomposite showed the best degradation efficiency.

## Introduction

However, as we learn more about how finite the earth’s supply of fossil raw materials has sparked a flurry of initiatives to discover environmentally friendly and long-term replacements. Aside from attempting to employ more renewable as a fundamental chemical feedstock, one of the key difficulties for science and engineering is meeting the world’s expanding energy demand^1^. As a result, it is critical to create new efficient technologies for converting energy from renewable sources. Hydrogen is one of the most interesting energy resources since it allows for the effective use of a fuel cell to convert chemical energy into electrical energy with the only byproduct being water^2^. As a result, an obvious benefit of the enormous reduction in pollutants is a hydrogen economy.

Due to its many benefits, such as its simplicity of use, high efficiency, and absence of secondary pollutants, a lot of focus has lately been placed on photocatalysis as one of the emerging advanced oxidation methods. It is widely recognized as method of treating wastewater that shows the greatest promise as being eco-friendly to the environment^3,4^. Catalysts play a crucial part in the photocatalytic process, as we all know. Titanium dioxide, among the many photocatalysts, is of particular interest because of its chemical stability, peculiar physical characteristics, and lack of toxicity. It is frequently used in energy conversion, hydrogen production, air purification, and wastewater treatment^5^. Titanium dioxide, a semiconductor, helps electrons to go from the valence band to the conduction band when exposed to light.

Compared to more traditional chemical oxidation techniques, photocatalytic applications, and semiconductor-based photocatalysis in particular, stand out as the most attractive means for decomposing harmful substances to non-hazardous output. This is due to the fact that semiconductors are (i) cheap, (ii) are non-toxic, (iii) have a high surface area, (iv) have broad absorption spectra with high absorption coefficients, (v) exhibit tunable properties that can be modified through size reduction, doping, sensitizers, etc., (vi) afford the facility for multielectrons transfer process, and (vii) can be used for long periods of time without significantly losing its photocatalytic activity.

In order for a semiconductor to be photochemically active, its redox potential must be positive enough for the photogenerated valence band hole to produce adsorbed OH radicals that can oxidise organic pollutants and negative enough for the conduction band electron to reduce adsorbed O_2_ to superoxide.

**Fig. 1:**
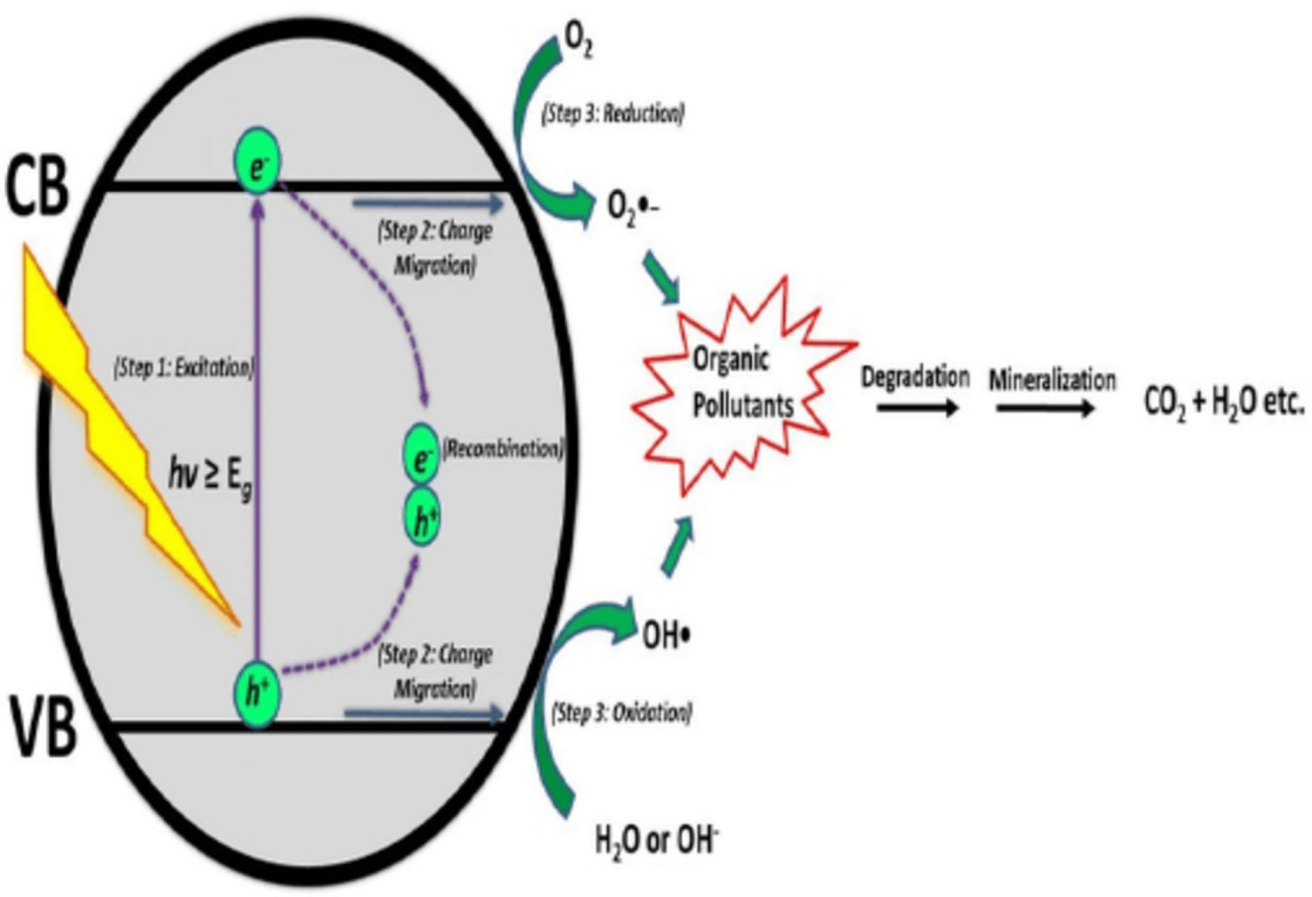
Schematic of the chemical reaction pathway of an n-type semiconductor such as TiO_2_.

The category of photocatalysts known as metal-oxide semiconductor photocatalysts includes TiO_2_. TiO_2_ is a good n-type semiconductor photocatalyst because of its high activity in Fig. 1, sustained resistance to photo- and chemical-corrosion during reaction circumstances, strong oxidizing power, stable substance, and nontoxic. TiO_2_nanostructures, on the other hand, are a potential semiconductor for a variety of applications, including batteries, solar cells, dye pollution catalysis, hydrogen generation, and water splitting^6–8^.

The three crystal phases of TiO_2_are rutile, brookite and anatase^9^. Although antase and rutile are the most commonly investigated phases, anatase has a greater photocatalytic degradation activity with organic pollutants than rutile due to its crystal structural arrangement^10^. TiO_2_ can only absorb ultraviolet light due to its high band gap energy 3.2 eV for anatase and 3.0 eV for rutile, respectively. This prevents TiO_2_from being used for visible light absorption^11^.

Tin oxide (SnO_2_) has sparked a lot of scientific interest. SnO_2_ is an outstanding optical and electrical material that, at room temperature, is an of n-type semiconductor that has a wide band gap (3.6 eV), unusual optical transparency, low resistivity, and a high theoretical specific capacity. The electron mobility in SnO_2_ is quite high (between 100 and 200 cm^2^ V^1^ s^1^), which suggests that photoexcited electron transport happens more quickly. SnO_2_ nanoparticles (SnO_2_ NPs) have high band gap energies, high stability, and unusual structural and optical properties. Another distinctive property of SnO_2_ NPs is the blue shift of the band edge transition energy. Furthermore, as the wide surface area serves to increase photocatalytic reaction sites, the small size of SnO_2_ NPs may be a contributing factor in the considerable degradation rate of the organic dye on as-produced SnO_2_NPs with diameters smaller than 10 nm and increases electron-hole separation efficiency^12^.

## Results and discussion

### HR-TEM of TiO2–SnO2 wt% nanocomposites

TiO_2_-SnO_2_ nanocomposite samples were examined with HR-TEM to investigate the homogenety and the morphology of the samples. Prior to the investigation, the samples were coated with gold using sputtering coater (model: S 150 B, Edwards High Vacuum Ltd., England).

**Fig. 2:**
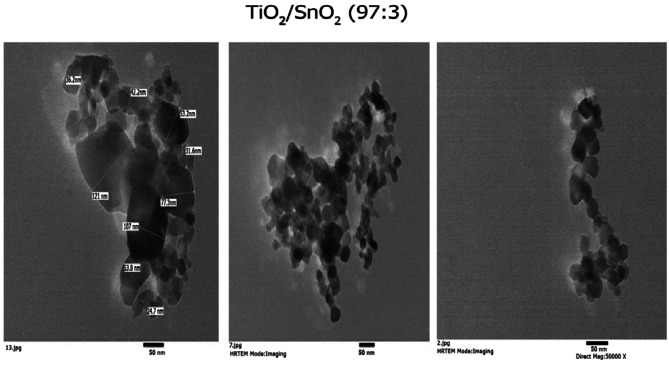
HR-TEM of TiO_2_–SnO_2_ (97:3) wt% nanocomposite.

Figure 2 shows the HR-TEM picture of the synthesized TiO_2_–SnO_2_ (97:3) wt% nanocomposite. The particles had tetragonal shape with a diameter of 26.3–88 nm, according to a detailed analysis of the micrograph.

**Fig. 3:**
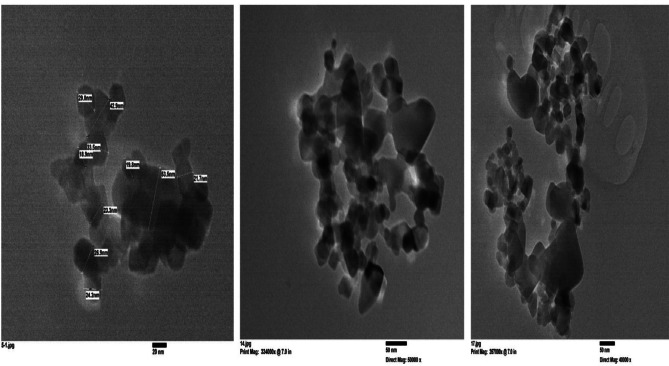
HR-TEM of TiO_2_–SnO_2_ (90:10) wt% nanocomposite.

Figure 3 shows the HR-TEM picture of the synthesized TiO_2_–SnO_2_ (90:10) wt% nanocomposite. The particles had a roughly tetragonal shape and semi round shape with a diameter of 27.6–54.5 nm, according to a detailed analysis of the micrograph.

**Fig. 4:**
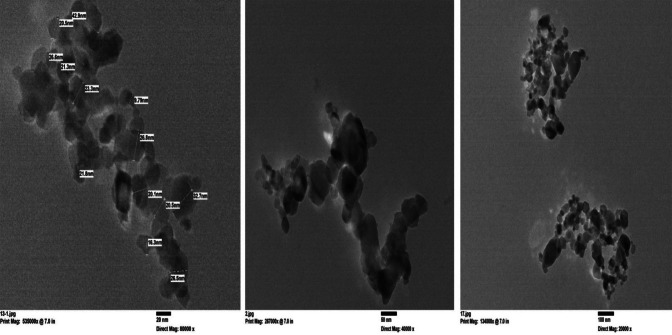
HR-TEM of TiO_2_–SnO_2_ (80:20) wt% nanocomposite.

Figure 4 shows the HR-TEM picture of the prepared TiO_2_–SnO_2_ (80:20) wt% nanocomposite. The particles had tetragonal and semi round shaped with a diameter of 30.2–72.2 nm, according to a detailed analysis of the micrograph.

It can be concluded from the previous Figs. 2, 3 and 4 that increasing of SnO_2_ (3 wt%) in TiO_2_–SnO_2_nanocomposite lead to an increase in the particle size due to nucleation and growth happen^13^. Further increase in SnO_2_ to 10 wt% results in increasing the particle size, whereas further increase in SnO_2_ up to 20 wt% results in decreasing the particle size of the nanocomposite due to agglomeration.

### XRD of the prepared TiO2–SnO2 nanocomposites

The purity, crystallite size, and crystal structure of the generated nanoparticles are all examined using XRD analysis. The width of a certain XRD peak in a diffraction pattern connected with a particular planar reflection inside the crystal unit cell is used to calculate crystallite size. The size of the crystallites is therefore reflected in the peak broadening.

#### XRD of TiO2–SnO2 (97:3) wt% nanocomposite

**Fig. 5:**
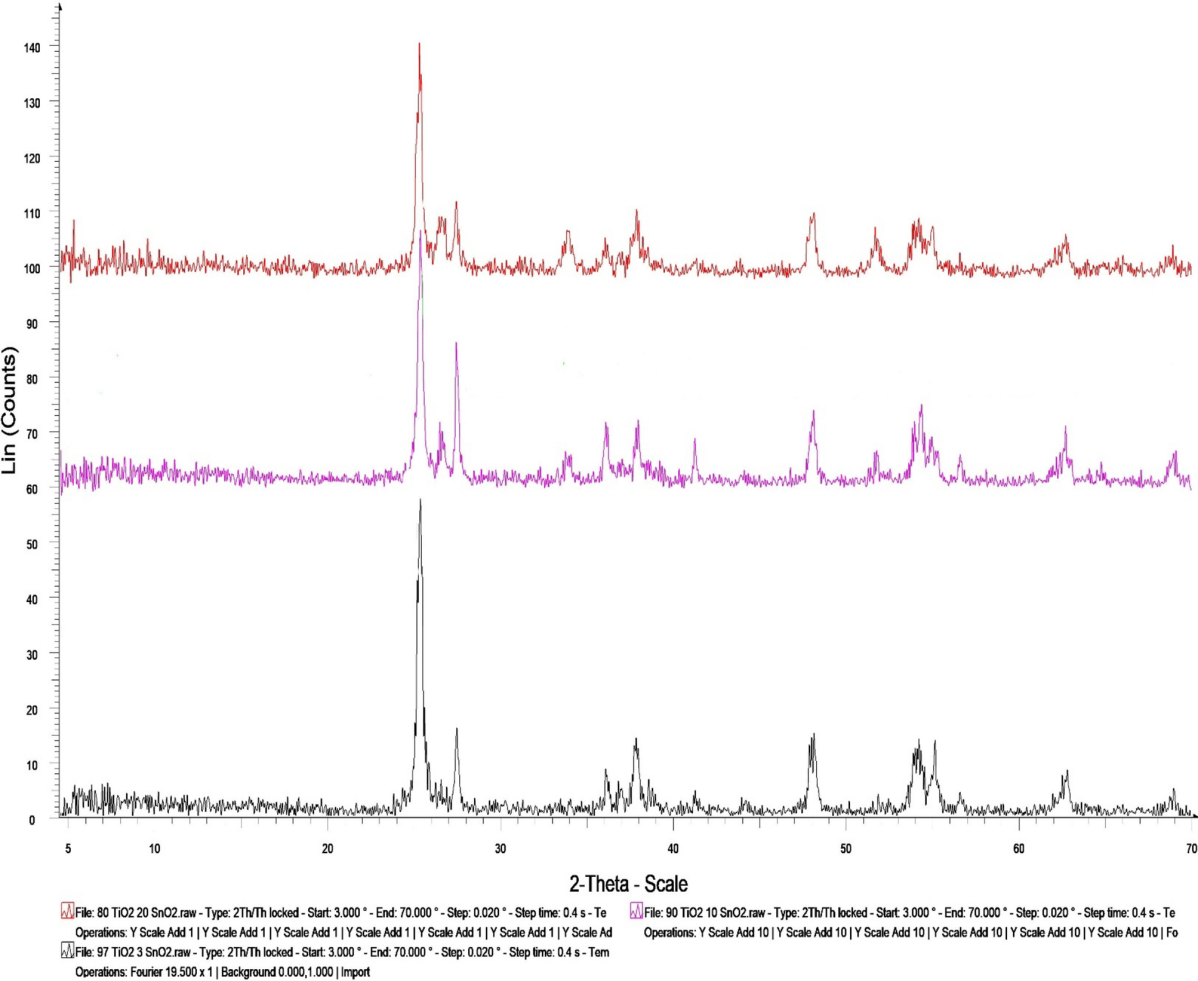
XRD of the prepared TiO_2_–SnO_2_ nanocomposites.

Figure 5 illustrates the XRD pattern of TiO_2_–SnO_2_ (97:3) wt% nanocomposite sample with diffraction peaks at 2*θ* = 25.30, 26.56, 27.42, 33.92, 36.09, 37.81, 38.65, 41.29, 47.99, 51.85, 54.03, 55.0, and 68.92. The crystal tetragonal structure’s allocated pattern, which indicates how it was formed, corresponds to TiO_2_–SnO_2_ (97:3) wt%. The measured diffraction peaks are consistent having a standard card issued by the Joint Committee of Powder Diffraction Standards (JCPDS card No 041-1445 and 001-0562) which showed a slight peak shift and an increase in the intensity of peak, with an average crystal size of 42.3 nm.

#### XRD of TiO2–SnO2 (90:10) wt% nanocomposite

Figure 5 depicts the XRD pattern of TiO_2_–SnO_2_ (90:10) wt% nanocomposite sample with diffraction peaks at 2*θ* = 25.33, 26.66, 27.43, 33.82, 36.08, 37.92, 41.24, 48.08, 51.75, 54.33, 56.68, 62.72, and 68.92. The tetragonal crystal structure’s allocated pattern, which indicates how it was formed corresponds to TiO_2_–SnO_2_ (90:10) wt%. The measured diffraction peaks are consistent having a standard card issued by the Joint Committee of Powder Diffraction Standard (JCPDS card No 041-1445 and 001-0562) which showed a slight peak shift and an increase in the intensity of peak, with an average crystal size of 37.09 nm.

#### XRD of TiO2–SnO2 (80:20) wt% nanocomposite

Figure 5 depicts the XRD pattern of TiO_2_–SnO_2_ (80:20) wt% nanocomposite sample with diffraction peaks at 2*θ* = 25.28, 26.57, 27.39, 33.87, 36.92, 35.92, 37.78, 41.29, 48.09, 51.70, 54.14, 62.77, and 68.86. The tetragonal crystal structure’s allocated pattern, which indicates how it was formed corresponds to TiO_2_–SnO_2_ (80:20) wt%. The measured diffraction peaks are consistent with the Joint Committee of Powder Diffraction standard (JCPDS card No 041-1445 and 001-0562) which showed a slight peak shift and an increase in the intensity of peak, with an average crystal size of 38.5 nm.

According to the results as shown Fig. 5 which obtained from XRD of TiO_2_-SnO_2_ nanocomposites, it could be concluded that the average crystal size by Scherrer Eq. (1) of TiO_2_–SnO_2_ (97:3) wt% was 42.3 nm and was found to decrease to 37.05 with increasing the w % of SnO_2_. Further increase of SnO_2_ wt% to 20 resulted in decreasing the average crystal size to 38.5 nm.

1 $$B(2\theta )=\frac{{K\lambda }}{{L\cos \theta }}$$

Peak width (B) is inversely proportional to crystallite size (L).

#### K (the Scherrer constant)—λ wavelength

**Fig. 6:**
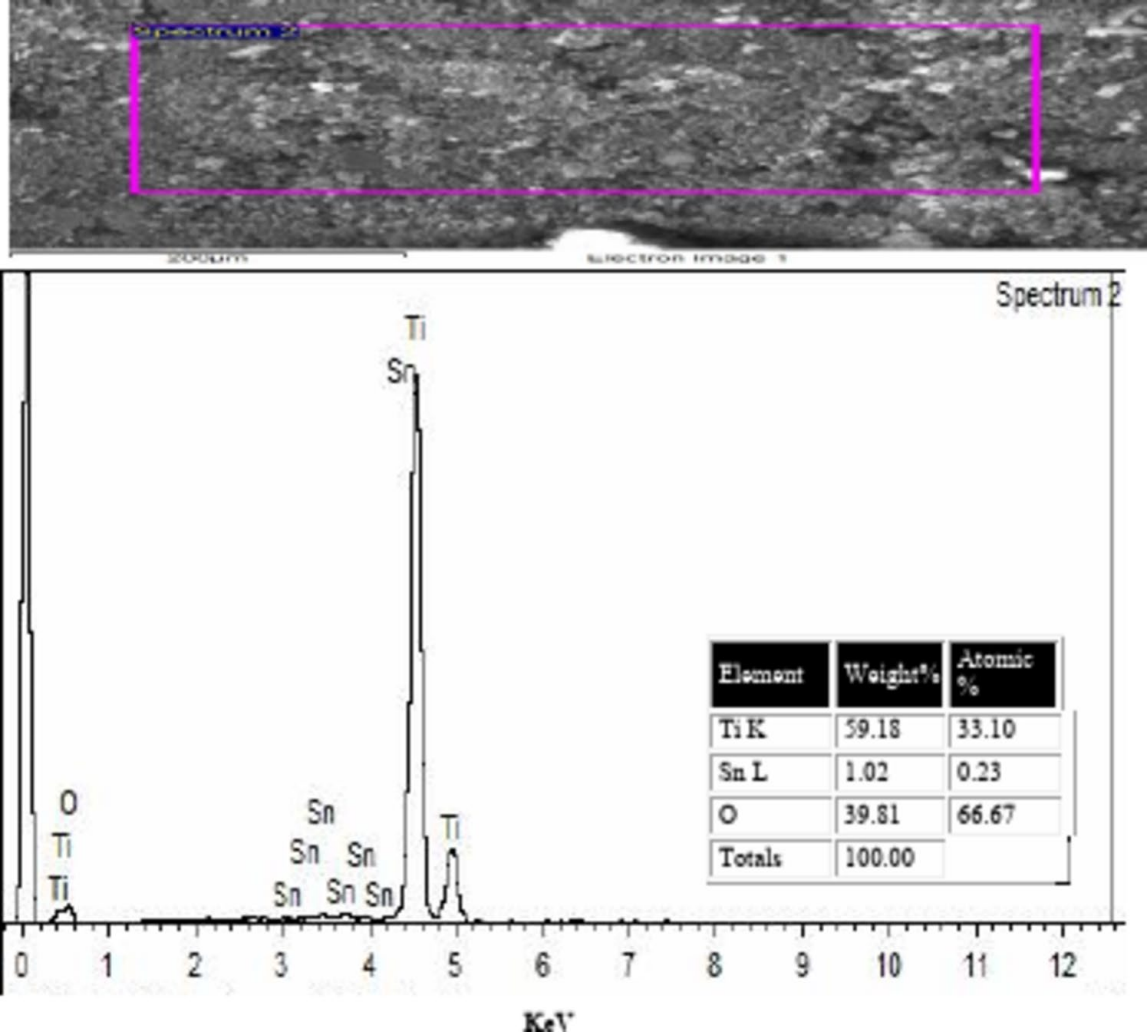
EDAX of TiO_2_–SnO_2_ (97:3) wt% nanocomposite.

**Fig. 7:**
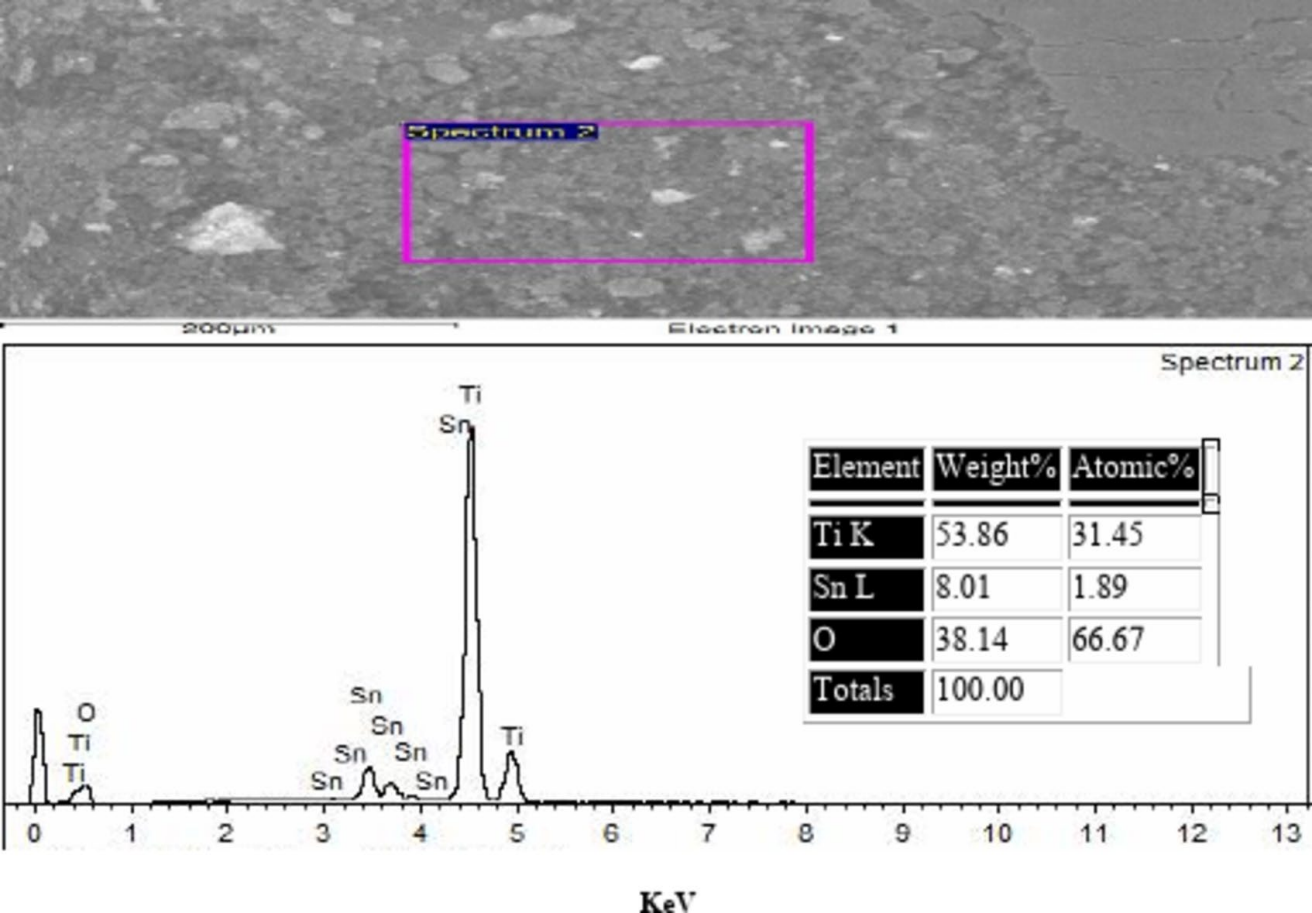
EDAX of TiO_2_–SnO_2_ (90:10) wt% nanocomposite.

According to the results as shown Figs. 5, 6 and 7 which obtained from XRD of TiO_2_-SnO_2_ nanocomposites, it could be concluded that the average crystal size of TiO_2_–SnO_2_ (97:3) wt% was 42.3 nm and was found to decrease to 37.05 with increasing the wt% of SnO_2_. Further increase of SnO_2_ wt% to 20 resulted in decreasing the average crystal size to 38.5 nm. respectively according to Table 1.

**Table 1:** Average crystal size (nm) of the prepared TiO_2_-SnO_2_ nanocomposites.

TiO_2_–SnO_2_ nanocomposite (wt%)	Average crystal size (nm)
(97:3)	42.3
(93:7)	41.1
(90:10)	37.05
(85:15)	40.9
(80:20)	38.5

### EDAX of the prepared TiO2–SnO2 nanocomposites

**Fig. 8:**
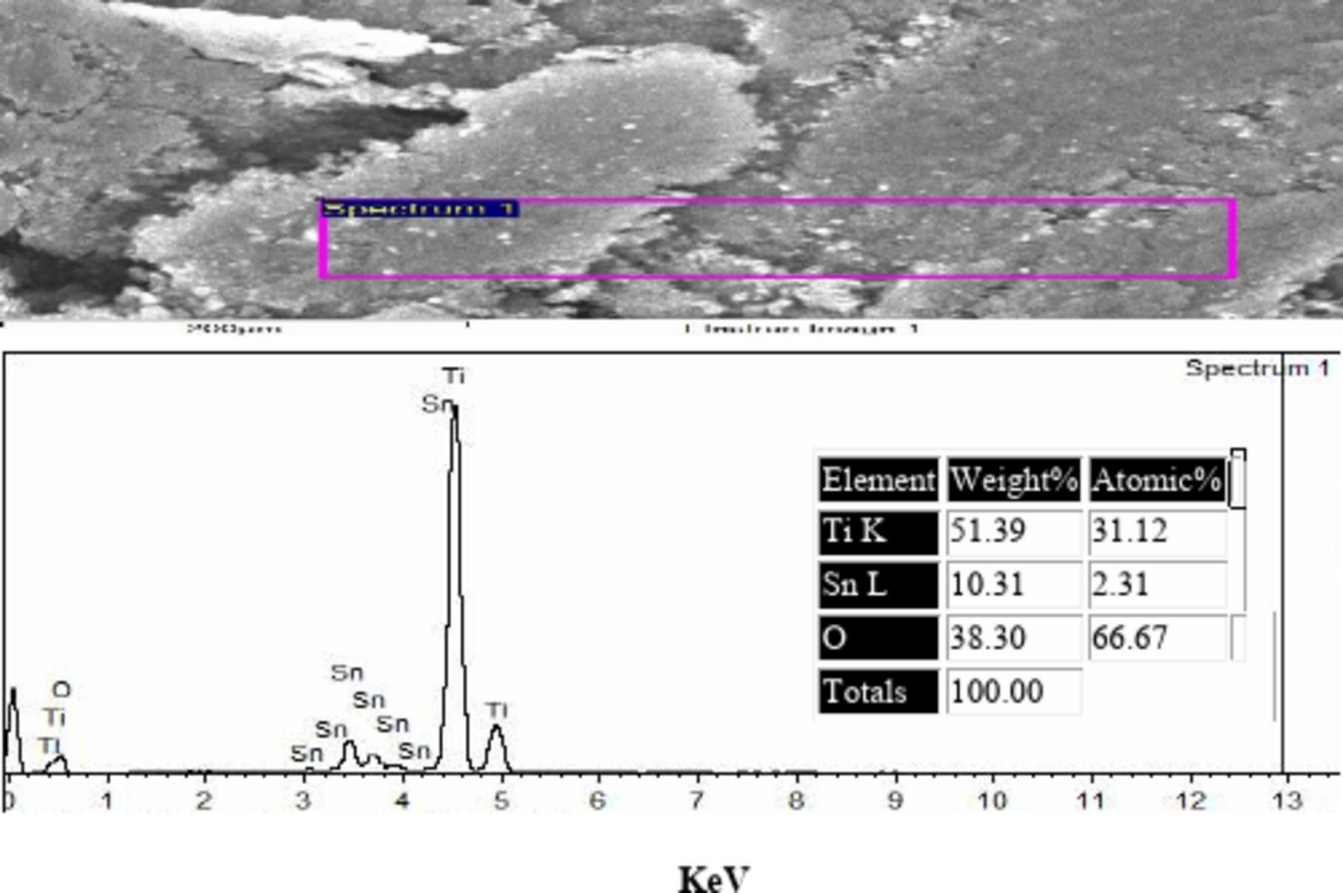
EDAX of TiO_2_–SnO_2_ (80:20) wt% nanocomposite.

The obtained results from the EDAX of TiO_2_–SnO_2_ nanocomposites as shown in Figs. 6, 7 and 8 confirmed the elemental ratios of the prepared nanocomposites.

### FTIR of the prepared TiO2–SnO2 nanocomposites

**Fig. 9:**
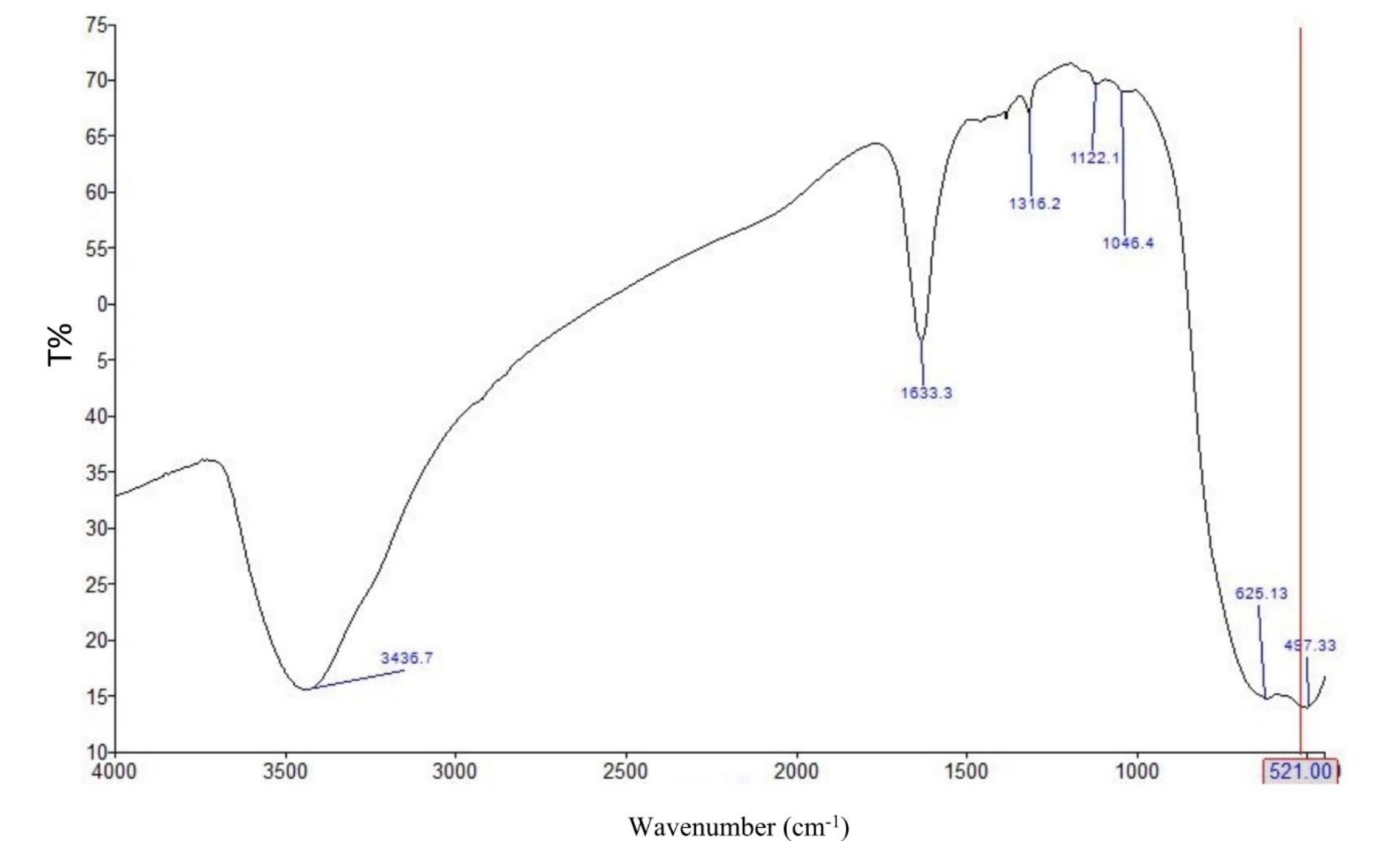
FTIR of TiO_2_–SnO_2_ (97:3) wt% nanocomposite.

**Fig. 10:**
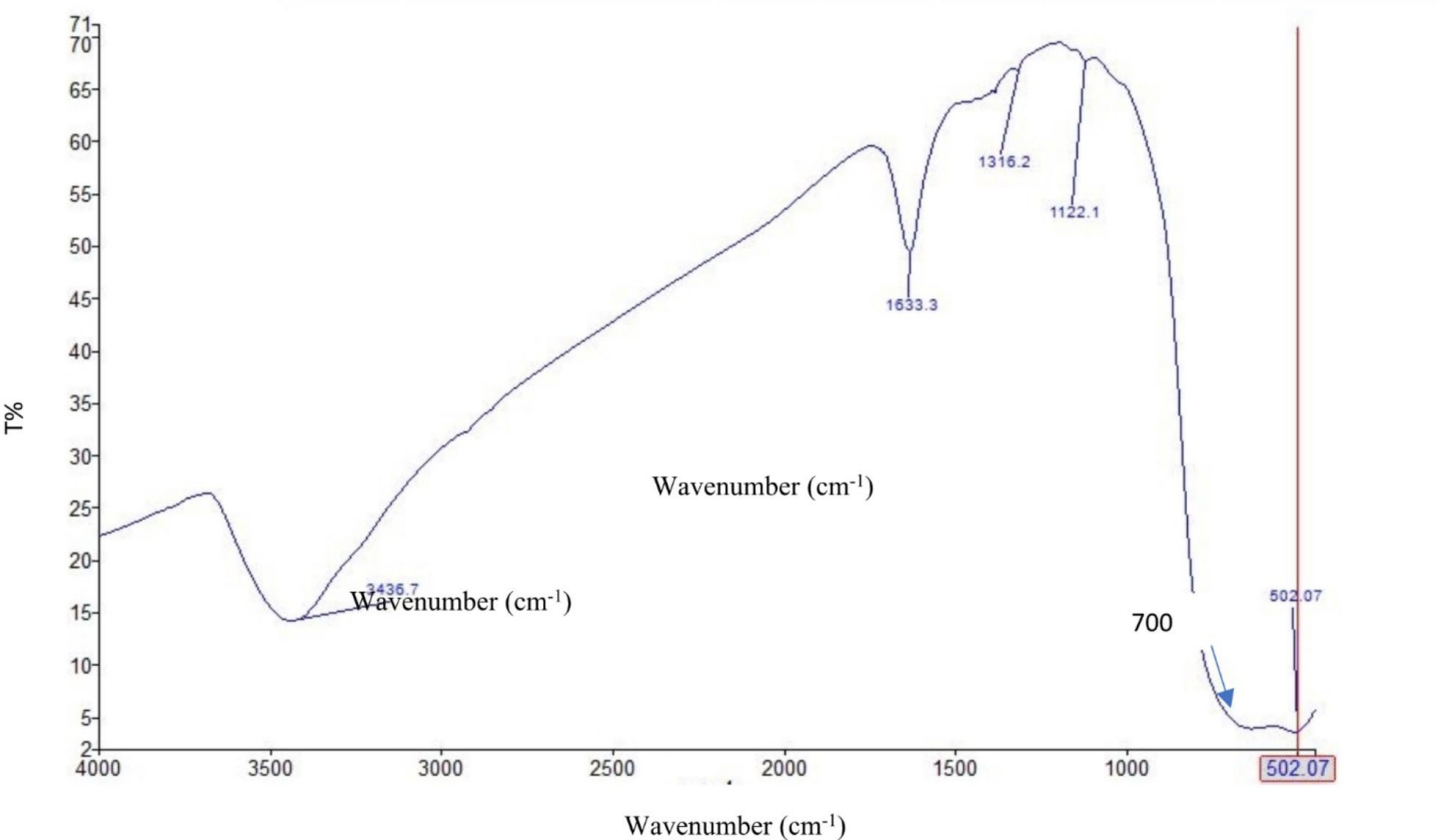
FTIR of TiO_2_–SnO_2_ (90:10) wt% nanocomposite.

**Fig. 11:**
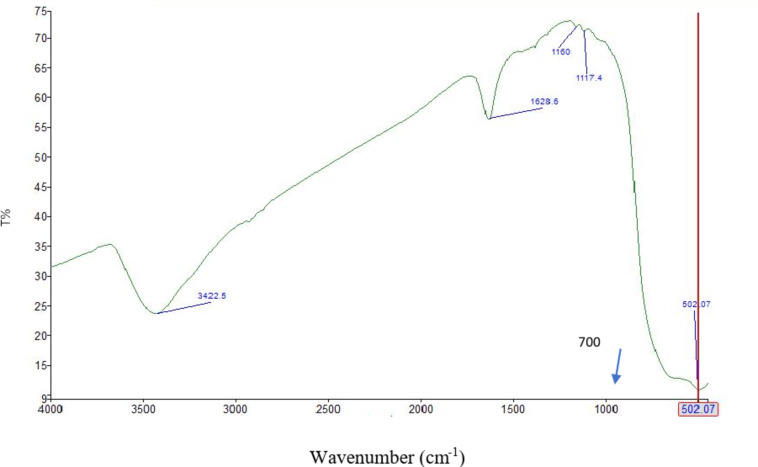
FTIR of TiO_2_–SnO_2_ (80:20) wt% nanocomposite.

As shown from Figs. 9, 10 and 11 the FTIR of TiO_2_–SnO_2_ nanocomposites shows that the peaks of TiO_2_ & SnO_2_ were slightly shifted due to the change in SnO_2_ loading percentage.

### XPS of the prepared TiO2–SnO2 nanocomposites

**Fig. 12:**
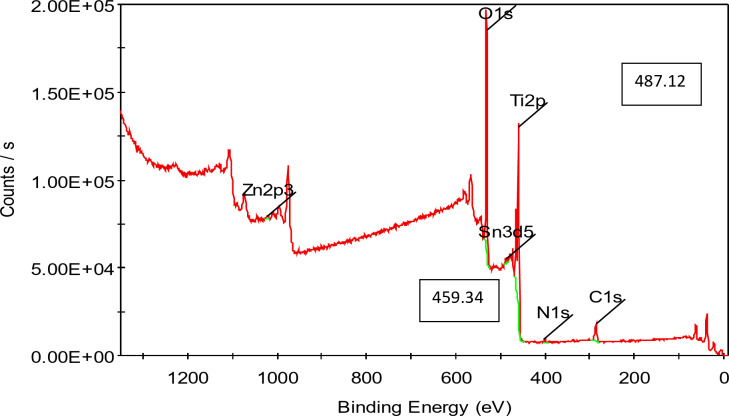
XPS of TiO_2_–SnO_2_ (90:10) wt% nanocomposite calcined at 500 °C for 3 h (Survey spectra).

**Fig. 13:**
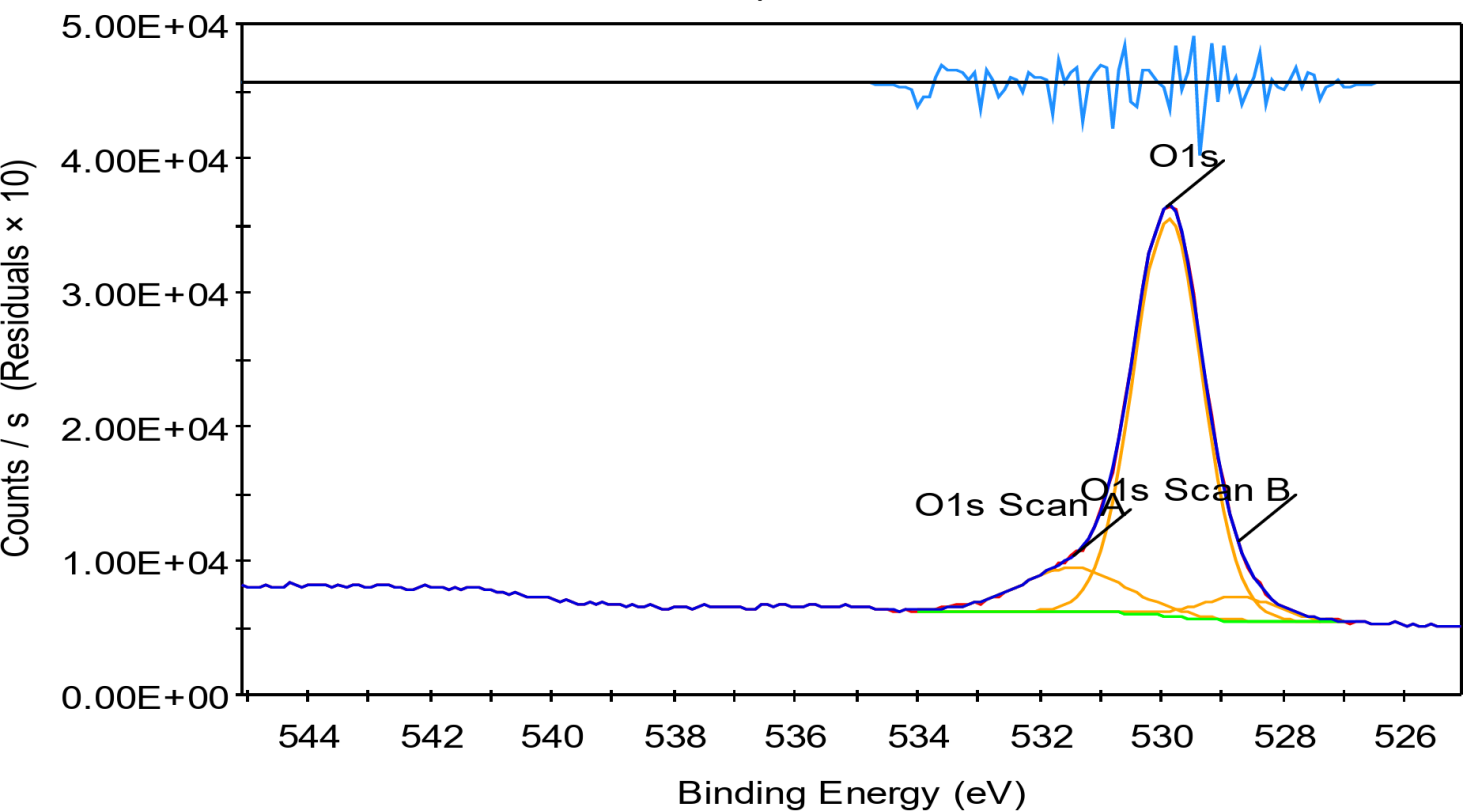
XPS of TiO_2_–SnO_2_ (90:10) wt% nanocomposite calcined at 500 °C for 3 h (O 1s region).

The analytical technique (XPS), commonly known as electron spectroscopy for chemical analysis (ESCA), that uses X-rays to bombard a specimen and then analyses the energy of the released electrons (Figs. 12 and 13).

#### XPS of TiO2–SnO2 (90:10) wt% nanocomposite

**Fig. 14:**
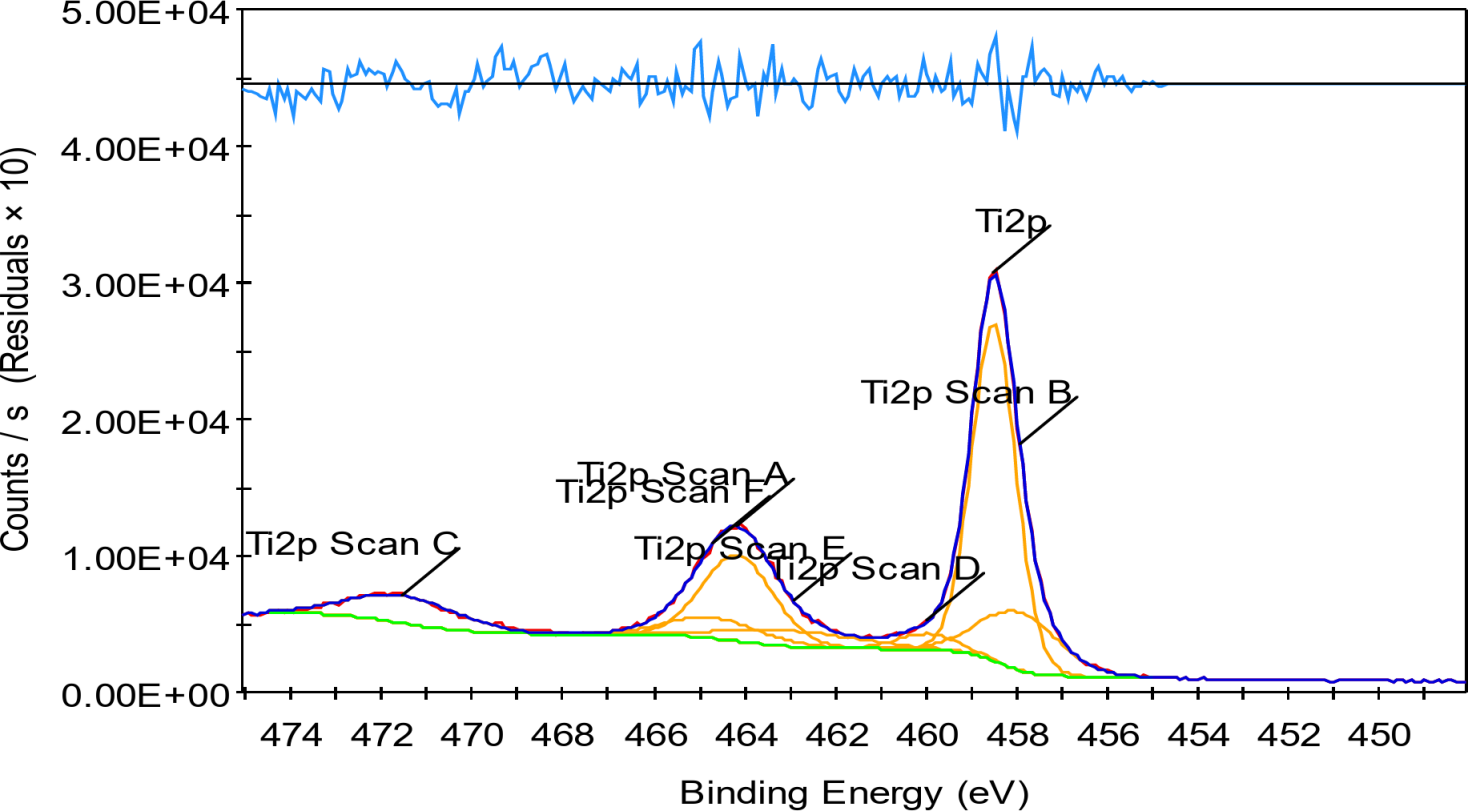
XPS of TiO_2_–SnO_2_ (90:10) wt% nanocomposite calcined at 500 °C for 3 h (Ti 2p region).

**Fig. 15:**
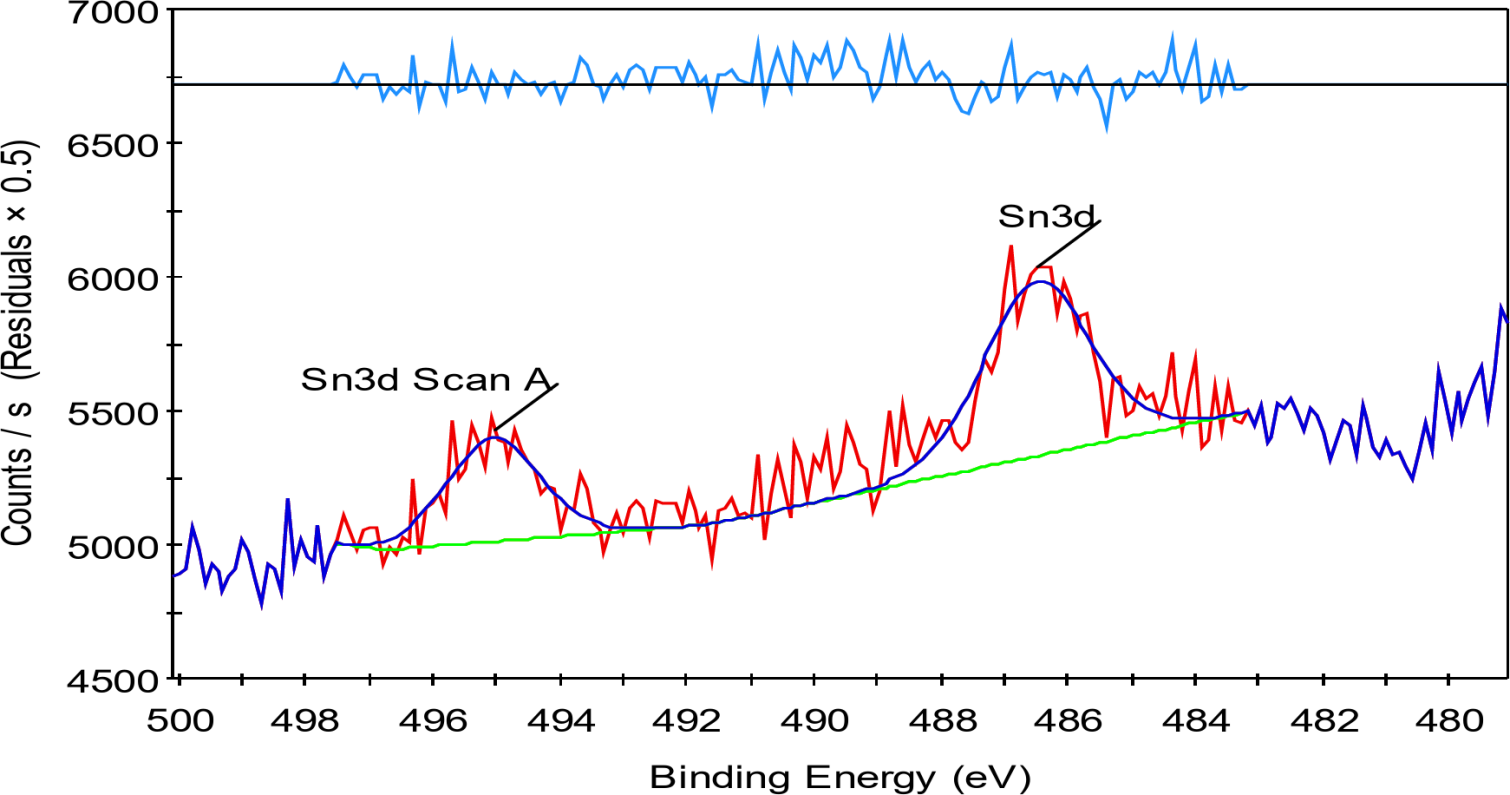
XPS of TiO_2_–SnO_2_ (90:10) wt% nanocomposite calcined at 500 °C for 3 h (Sn 3d region).

**Fig. 16:**
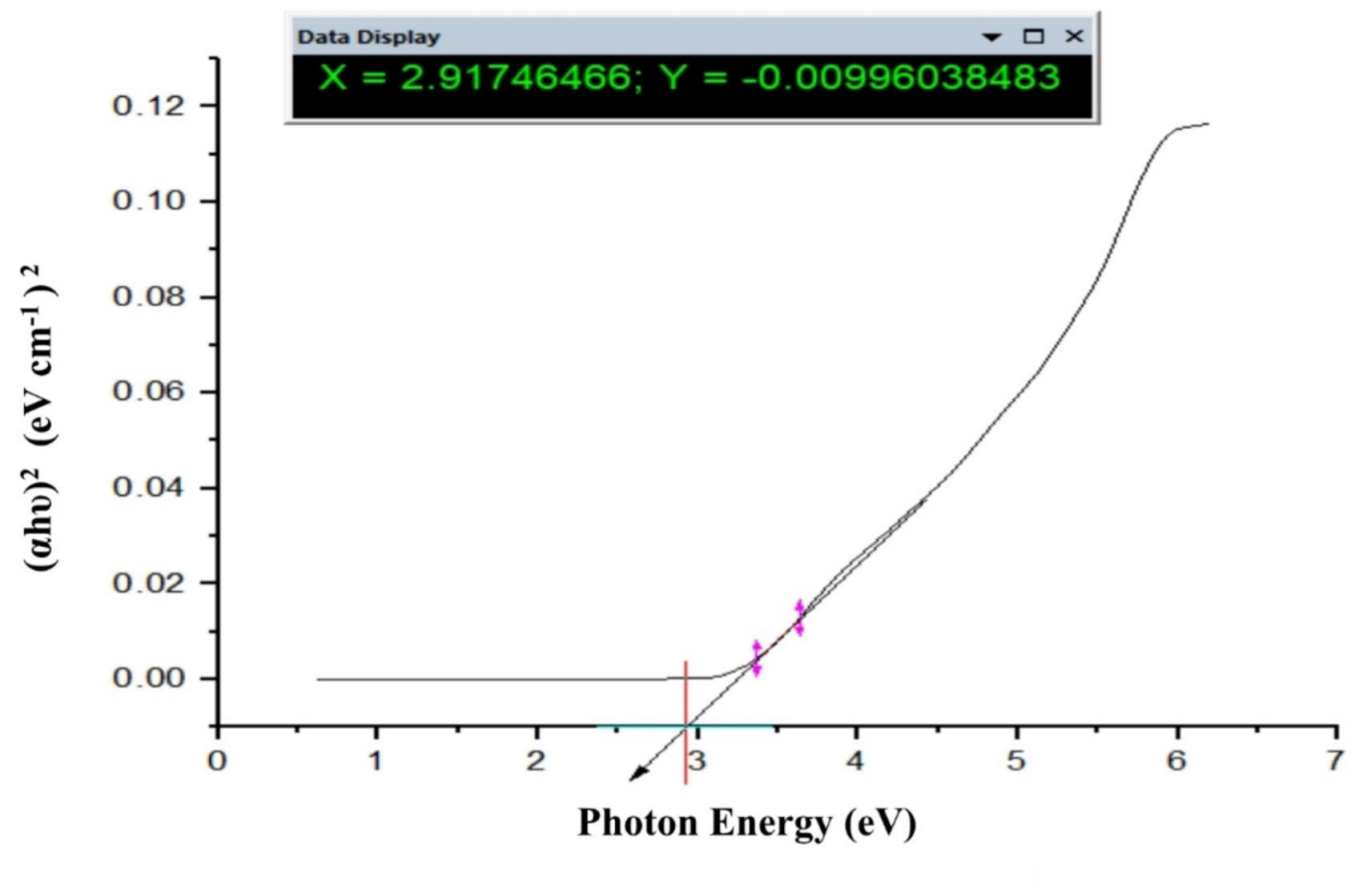
The band gap energy of TiO_2_–SnO_2_ (97:3) wt% nanocomposite.

**Fig. 17:**
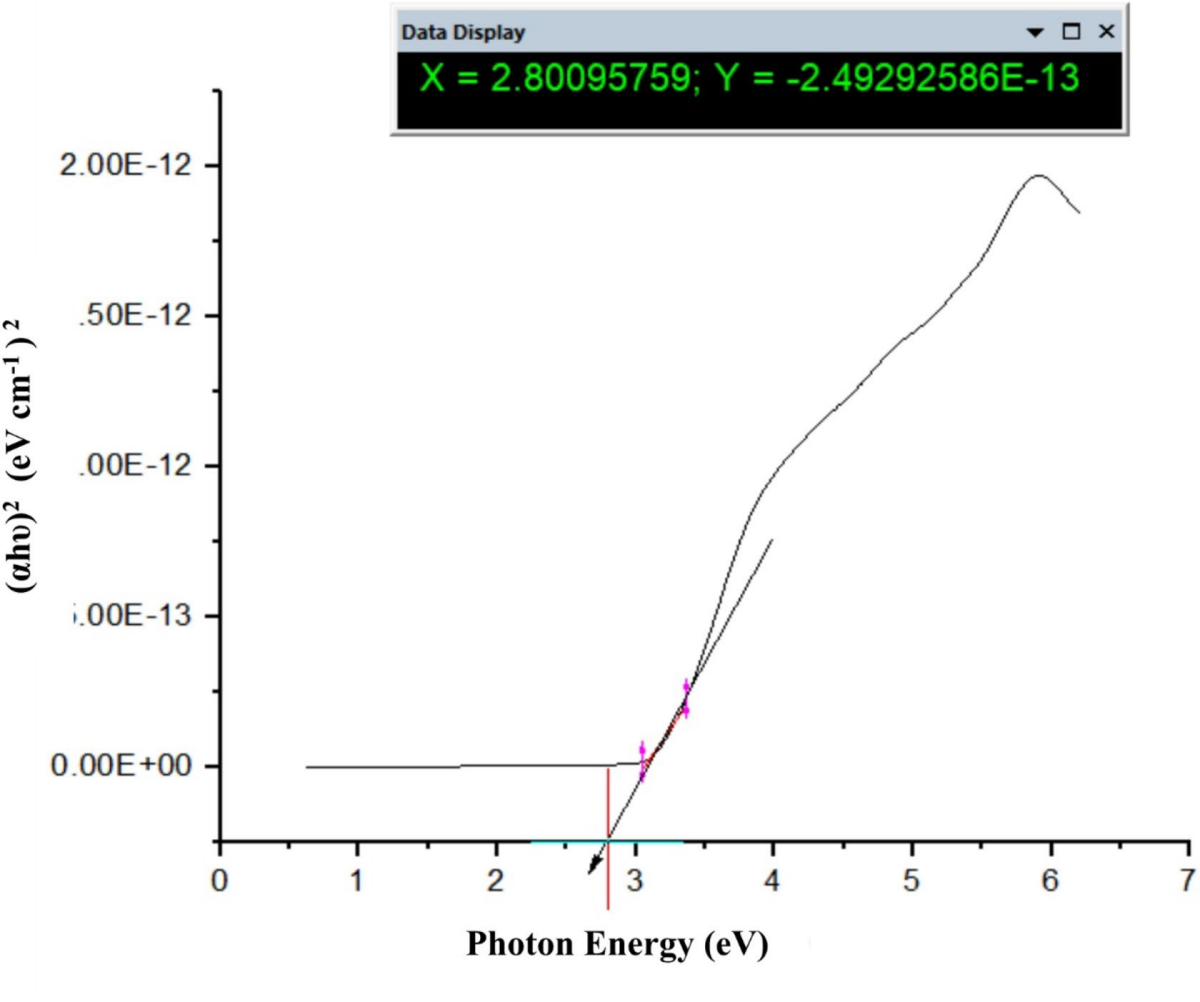
The band gap energy of TiO_2_–SnO_2_ (90:10) wt% nanocomposite.

Figures 14, 15, 16 and 17 shows the results of XPS analysis of the elements’ chemical make up and content. Peaks of O 1s, Ti 2p, Sn 3d, and C1s were seen in the survey spectra. Figure 14 in the region of 1200-0 eV, showing that Ti, O made up the majority of the samples. Figure 15 displays the spectrum of asymmetric TiO_2_-SnO_2_ (90:10) wt% with an O 1s core level. The spectrum was fitted by two Gaussian peaks at around 531.44 eV (Ti-O-Sn) and 528.75 eV. (Sn–O). Although there were still some Tin ions dispersed on the surface of TiO_2_, it was hypothesized that Ti-O-Sn bonds connecting the composite sample were generated it in the majority. This increased the oxygen concentration on the surface. It could be fitted into 6 peaks in the spectrum in Ti 2p region (Fig. 16) with energies of 464.15, 457.94, 471.54, 459.98, 462.91, and 464.69 eV. 464.15 and 457.94 eV peaks were attributed to Ti^4+^2p_1/2_ and Ti^4+^2p_3/2_, respectively. Figure 17 displays the doublet Sn 3d spectrum, with binding energies of 495.03 and 486.49 eV, respectively, for Sn^3+^3d_3/2_ and Sn^3+^3d_5/2_lines^14^.

Finally, from the previous Figs. 12, 13, 14 and 15 it was found that the catalysts might have more lattice defects. The presence of Sn^3+^, could make it easier for electrons to be captured. As a result, the likelihood of photoinduced electrons and holes recombining was decreased^14^.

### Calculations of band gap energy of TiO_2_–SnO_2_nanocomposites

#### Calculation of band gap energy of TiO_2_–SnO_2_(97:3) wt% nanocomposite

It was found that, increasing the amount of SnO_2_ in TiO_2_–SnO_2_ nanocomposite causes a red-shift to longer wavelengths. This has to do with the development of new energy levels in TiO_2_band gap^6,15^. Oxygen vacancies are the principal source of the tiny absorption edges in the visible area^16,17^.

**Fig. 18:**
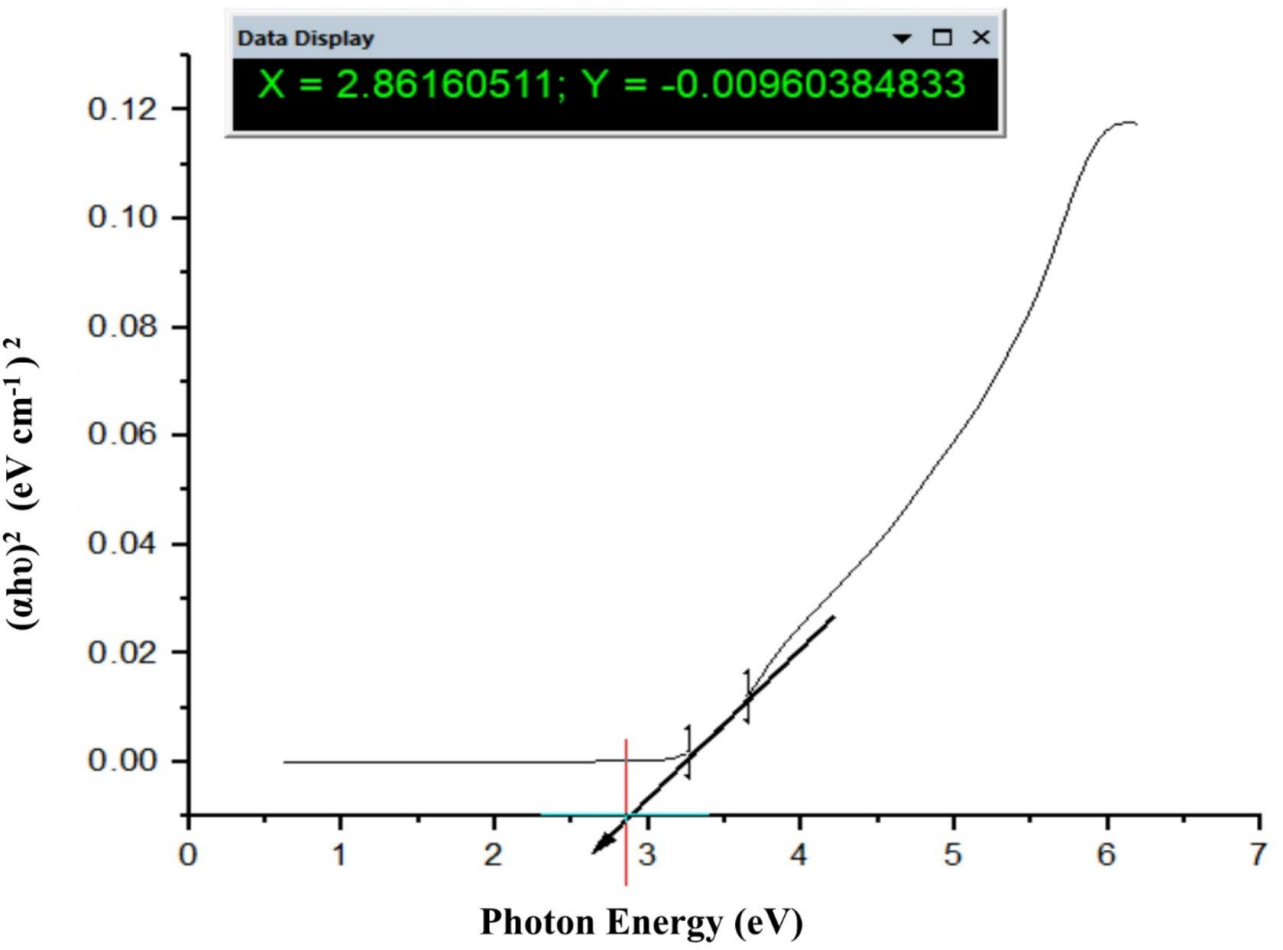
The band gap energy of TiO_2_–SnO_2_ (80:20) wt% nanocomposite.

The UV-Vis analysis is applied to investigate and calculate the band gap energy by using Tauc Eq. (1).

1 $$\alpha {\text{h}}\upsilon \,=\,{\text{A}}{({\text{h}}\upsilon - {\text{Eg}})^{\text{n}}},$$

where *n* = 1/2 for direct band gap materials and *n* = 2 for indirect band gap materials, α is absorption coefficient, h is the Planck constant, υ is the wavenumber, A is a constant, and E_g_is the band gap energy^18^. The band gap energy (E_g_) value of TiO_2_–SnO_2_ (97:3) wt% is determined from the plot of (αhυ)^2^ against photon energy in electron volts. The derived band gap energy from Figs. 16, 17 and 18 was obtained from the slope of the line. The calculated band gap energy of TiO_2_–SnO_2_ (97:3) wt% nanccomposite was found to be 2.91 eV which was a less value compared with TiO_2_ (3.2 eV) and SnO_2_(3.6 eV)^19^.

Figure 17 depicts the band gap energy (E_g_) of TiO_2_–SnO_2_ (90:10) wt% nanocomposite. The band gap energy (E_g_) of TiO_2_–SnO_2_ (93:7) wt% is determined from the plot of (αhυ)^2^ against photon energy in electron volts which was found to be 2.8 eV when compared with TiO_2_ (3.2 eV) and SnO_2_ (3.6 eV). So, it is decreased with increasing the wt% of SnO_2_in the nanocomposite^19^.

Figure 18 depicts the band gap energy (E_g_) of TiO_2_–SnO_2_ (80:20) wt% nanocomposite. The calculated band gap energy was found to be 2.86 eV. So, it is increased again with increasing the wt% of SnO_2_to 20 in the nanocomposite^19^.

From the previous results on band gap calculation, it was found according to band gap values as shown in Table 2 that the prepared nanocomposites have lower band gap energies than native TiO_2_ and SnO_2_.

When SnO_2_ wt% increases from (3–10), the band gap decreases from 2.91 to 2.8 eV and then return to increase when SnO_2_ wt% increases from (15–20). This may be due to band gap energy of the coupled oxides should results from band gap overlap with the individual oxides. The band gap was found to shrink slightly as Sn concentration increased. Reportedly, SnO_2_ exhibits a narrow indirect band gap (3.4 eV) and a wide direct band gap (< 3.4 eV) (1.6 to 2.9 eV). The shift in the band gap energy with increasing the wt% of SnO_2_ is likely due to changes in crystal shifting on the plane, as indicated by XRD.

Also, doping of TiO_2_ with SnO_2_ creates Fermi level below the conduction band of the nanocomposite, so the band gap of TiO_2_–SnO_2_ nanocomposite decreases compared with native TiO_2_ and SnO_2_alone^20^.

Also, it was found that increasing the percentage of SnO_2_ from 3 to 10 wt% resulted in decreasing the Fermi level position and hence decreasing the band gap energy of TiO_2_-SnO_2_ nanocomposite from 2.91 to 2.8 eV. Further increasing in the wt% of SnO_2_ from 15 to 20 resulted in increasing the band gap from 2.82 to 2.86 eV respectively. This increase in the band gap energy is probably due to the formation of homojunction of SnO_2_–SnO_2_ on the expense of TiO_2_ -SnO_2_ nanocomposite. So, the prepared nanocomposites need lower energy for electron excitation from VB to CB than native TiO_2_ and SnO_2_.

It was concluded also that TiO_2_-SnO_2_ (90:10) wt% nanocomposite has the lowest band gap value (2.8 eV), so (90:10) wt% is the optimum ratio which means it needs lower energy for excitation as shown in Table 2.

**Table 2:** Effect of SnO_2_ percentage on TiO_2_-SnO_2_ nanocomposite band gap energy.

Sample	Band gap energy (eV)
TiO_2_	3.2
SnO_2_	3.6
TiO_2_–SnO_2_ (97:3)	2.91
TiO_2_–SnO_2_ (93:7)	2.88
TiO_2_–SnO_2_ (90:10)	2.80
TiO_2_–SnO_2_ (85:15)	2.82
TiO_2_–SnO_2_ (80:20)	2.86

#### Photocatalysis of acid red 37 dye

The photocatalytic degradation of aqueous acid Red 37 dye solution was studied with different AOPs using UV/TiO_2_, UV/SnO_2_, UV/TiO_2_–SnO_2_ (97:3), UV/TiO_2_–SnO_2_ (90:10), UV/TiO_2_–SnO_2_ (80:20), UV/TiO_2_–SnO_2_ (90:10)/H_2_O_2_, UV/TiO_2_–SnO_2_ (90:10)/S_2_O_8_ and UV/TiO_2_–SnO_2_ (90:10)/IO_4_^−^. In every experiment, the batch photoreactor was employed as the photoreactor.

#### Effect of native TiO2, SnO2 and TiO2–SnO2 nanocomposites as photocatalysts

Photodegradation was caused when UV light with a wavelength of 254 nm was used to irradiate slurry solutions of TiO_2_ and SnO_2_ (0.4 g L^−1^) containing acid red 37 dye. This can be represented as shown in Fig. 1as mentioned in the introduction^1,21–23^. A first order model for the degradation of dye was used to visualize the data. The slopes of the straight lines on plots of ln (C/C_o_) over time, where C_o_ represents the starting concentration of acid red 37 dye and C is the dye concentration at time t, were used to calculate the values of the first-order rate constant (k_app_). The time necessary for the reactants to deteriorate to half of their starting concentrations is known as the half-life time (t_1/2_) of the pseudo first order reaction. t_1/2_ and k_app_ are inversely proportional as shown by Eq. (2).

2 $${{\text{t}}_{{\text{1}}/{\text{2}}}}={\text{ }}0.{\text{693}}/{{\text{k}}_{{\text{app}}}}.$$

It was discovered that increasing the apparent rate constant (k_app_) causes the t_1/2_ to decrease, as indicated in Table 3.

**Table 3:** The apparent rate constants and half life times for photocatalytic degradation of acid red 37 dye using UV/TiO_2_, UV/SnO_2_, UV/TiO_2_–SnO_2_ (97:3), UV/TiO_2_–SnO_2_ (90:10) and UV/TiO_2_–SnO_2_ (80:20).

Catalytic system	Catalyst loading (g/L)	k_app_ (min^−1^)	t_1/2_ (min)
UV/TiO_2_	0.4	2 × 10^−4^	346.0
UV/SnO_2_	0.4	28 × 10^−4^	247.1
UV/TiO_2_–SnO_2_ (97:3)	0.4	38 × 10^−4^	182.1
UV/TiO_2_–SnO_2_ (90:10)	0.4	72 × 10^−4^	96.1
UV/TiO_2_–SnO_2_ (80:20)	0.4	35 × 10^−4^	197.7

Irradiation of SnO_2_ (0.4 g L^−1^) with UV light of wavelength 254 nm resulted in a lower photodegradation of acid red 37 dye compared with UV/TiO_2_. But SnO_2_ exhibited good catalytic performance and stability. The superior photocatalytic activity of TiO_2_ over SnO_2_ may be attributed to smaller TiO_2_ particle size, quick e^−^/h^+^ pair recombination rate and poor quantum yield of SnO_2_in aqueous solutions^24^.

**Fig. 19:**
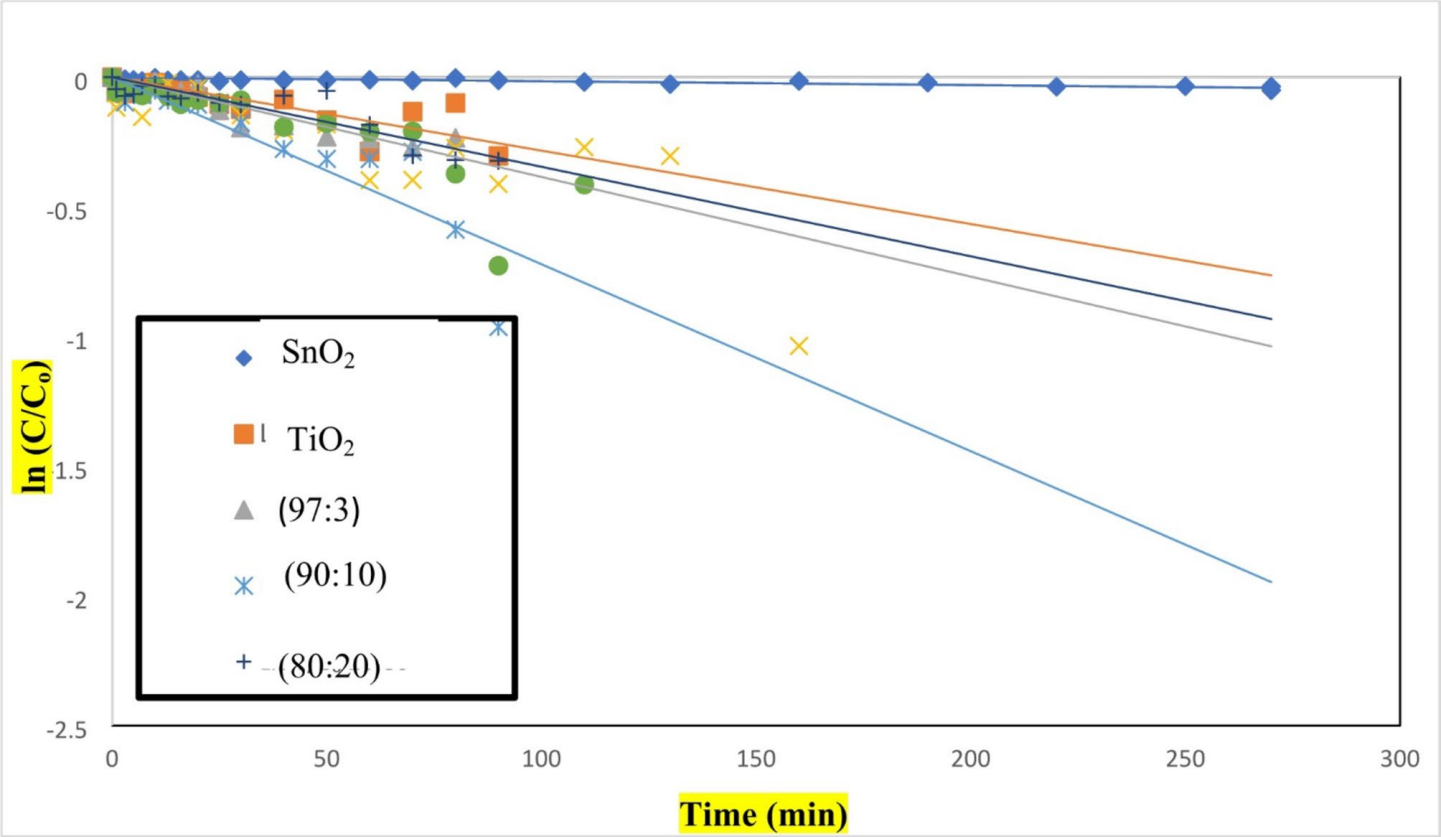
Change of ln (C/C_o_) with time for photocatalytic degradation of acid red 37 dye using UV/TiO_2_, UV/SnO_2_, UV/TiO_2_–SnO_2_ (97:3), UV/TiO_2_–SnO_2_ (93:7), UV/TiO_2_–SnO_2_ (90:10), UV/TiO_2_–SnO_2_ (85:15) and UV/TiO_2_–SnO_2_ (80:20).

According to Fig. 19, the synthesized TiO_2_–SnO_2_ nanocomposite exhibits a higher photocatalytic activity compared with native TiO_2_ or SnO_2_. TiO_2_–SnO_2_ composite’s high photocatalytic activity can be attributed to the more effective separation of photoinduced electron–hole (e^−^/h^+^) pairs; specifically, the n-p heterojunction of TiO_2_–SnO_2_ causes the photogenerated holes to migrate toward the interface and the electrons to migrate toward the bulk.

**Fig. 20:**
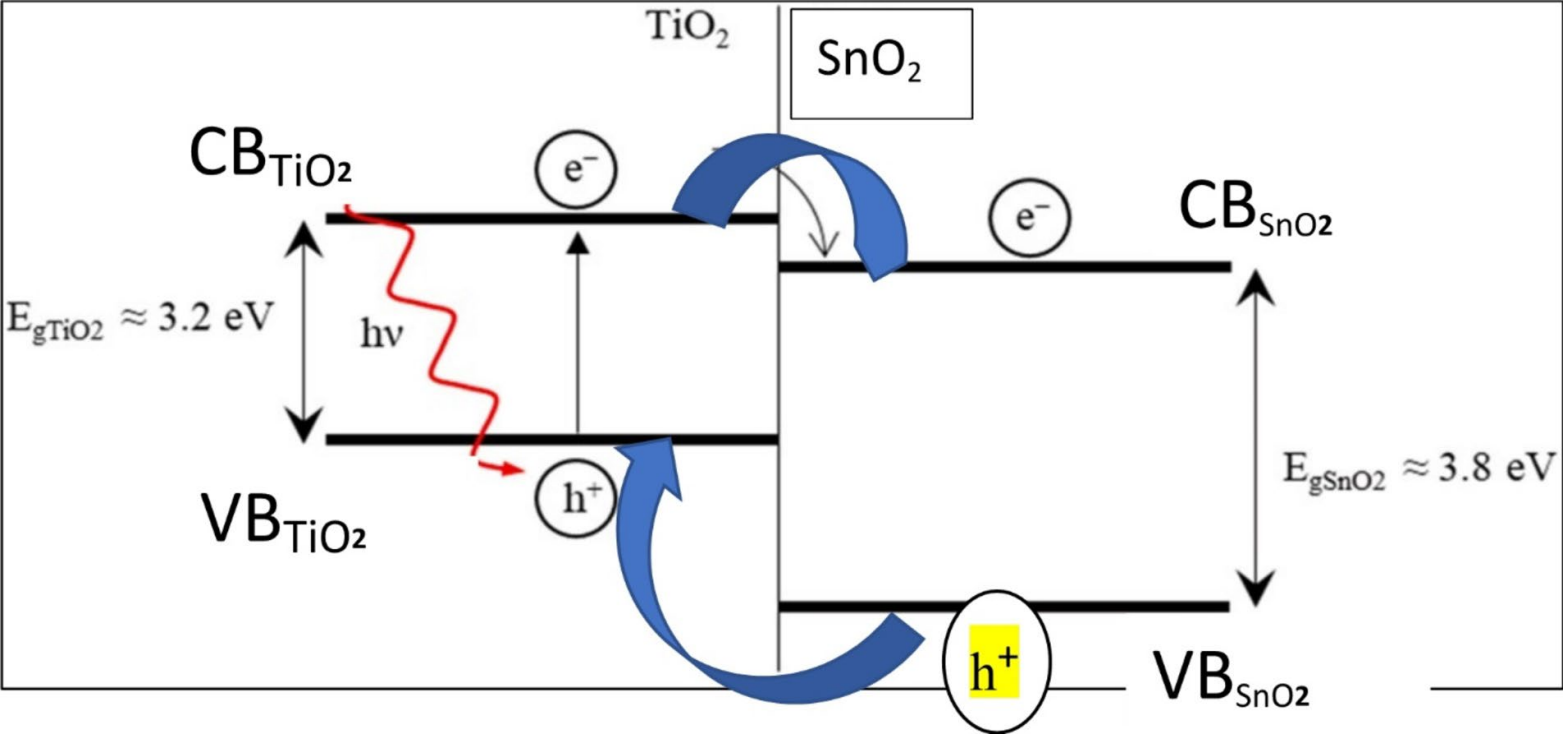
Relative energy position of the bands and the process of charge separation in theTiO_2_/SnO_2_ system.

Because the conduction band of SnO_2_ CB_SnO2_ is more positive than that of the conduction band of TiO_2_ CB_TiO2_, the excited electrons on TiO_2_ can also move to SnO_2_ whereas the photogenerated holes can also move to the valance band of SnO₂ loading VB_SnO2_ to the conduction band of TiO_2_ VB_TiO2_ (e^−^/h^+^) pairs separation as shown in Fig. 20. In comparison with native TiO_2_ or SnO_2_, TiO_2_–SnO_2_ nanocomposite demonstrated much better photocatalytic activity. Degradation of acid red 37 dye via photocatalysis depends significantly on the content of SnO_2_ present in TiO_2_–SnO_2_ nanocomposite. It was discovered that increasing the quantity of SnO_2_ from 3 to 10 wt% led to a stronger photocatalytic degradation of the dye. This may be due to decreasing in the band gap as shown in band gap calculation to a certain limit at TiO_2_–SnO_2_ (90:10) wt% nanocomposite, which is the lowest band gap that avoid the recombination and decreases the time needed to transfer the electrons and holes between CB and VB which gives the highest photocatalytic activity. The decreasing in photocatalytic degradation of the dye with increasing SnO_2_ content from 10 to 20 wt% may be due to the formation of SnO_2_–SnO_2_ homojunctions which decrease the number of TiO_2_–SnO_2_ heterojunctions and also increase the number of free SnO_2_particles, which possess low photocatalytic activity^25^.

### Effect of addition of various inorganic oxidants to TiO2–SnO2 (90:10) nanocomposite

#### Effect of addition of H2O2 to TiO2–SnO2 (90:10) wt% nanocomposite

**Fig. 21:**
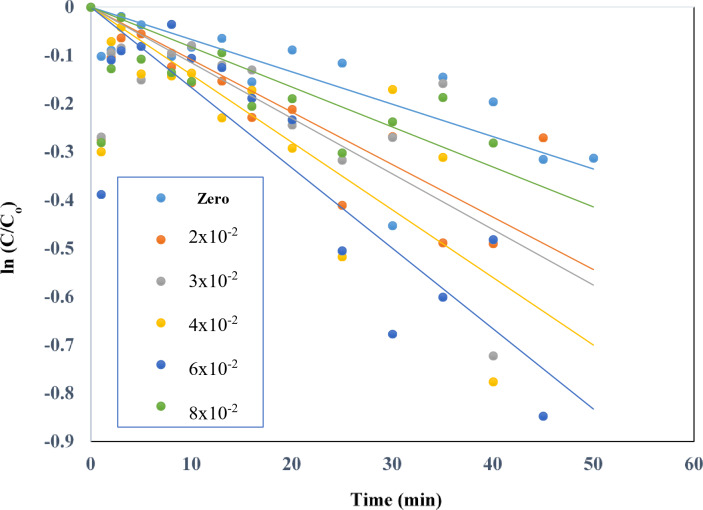
Change of ln (C/C_o_) with time for photocatalytic degradation of acid red 37 dye using 0.4 g/L of TiO_2_–SnO_2_ (90: 10) wt% with different concentrations of H_2_O_2_ (M).

TiO_2_–SnO_2_ (90: 10) wt% nanocomposite with SnO_2_ (10 wt%) was utilized to explore the influence of several oxidants (H_2_O_2_, Na_2_S_2_O_8_ and NaIO_4_) on the photocatalytic breakdown of acid red 37 dye based on earlier findings. Figure 21 depicts the effect of adding different concentrations of hydrogen peroxide to TiO_2_–SnO_2_ (90:10) (0.4 g L^−1^) for photodegradation of acid red 37 dye under UV irradiation^21^. The data was plotted using a first-order dye destruction model. The addition low concentration of hydrogen peroxide (2 × 10^−2^ M) to UV/TiO_2_-SnO_2_ (90:10) wt% (0.4 g L^−1^) increases the rate of photocatalytic degradation of acid red 37 dye when compared with UV/TiO_2_-SnO_2_ (90:10) wt% alone. The rate of photodegradation increased when the concentration of hydrogen peroxide was increased to 6 × 10^−2^ M with k_app_ ranged from 789 × 10^−4^− 997 × 10^−4^ min^−1^, then decreased with further increasing of hydrogen peroxide concentration to 8 × 10^−2^ M with k_app_ of 698 × 10^−2^ M (Table 1).

**Fig. 22:**
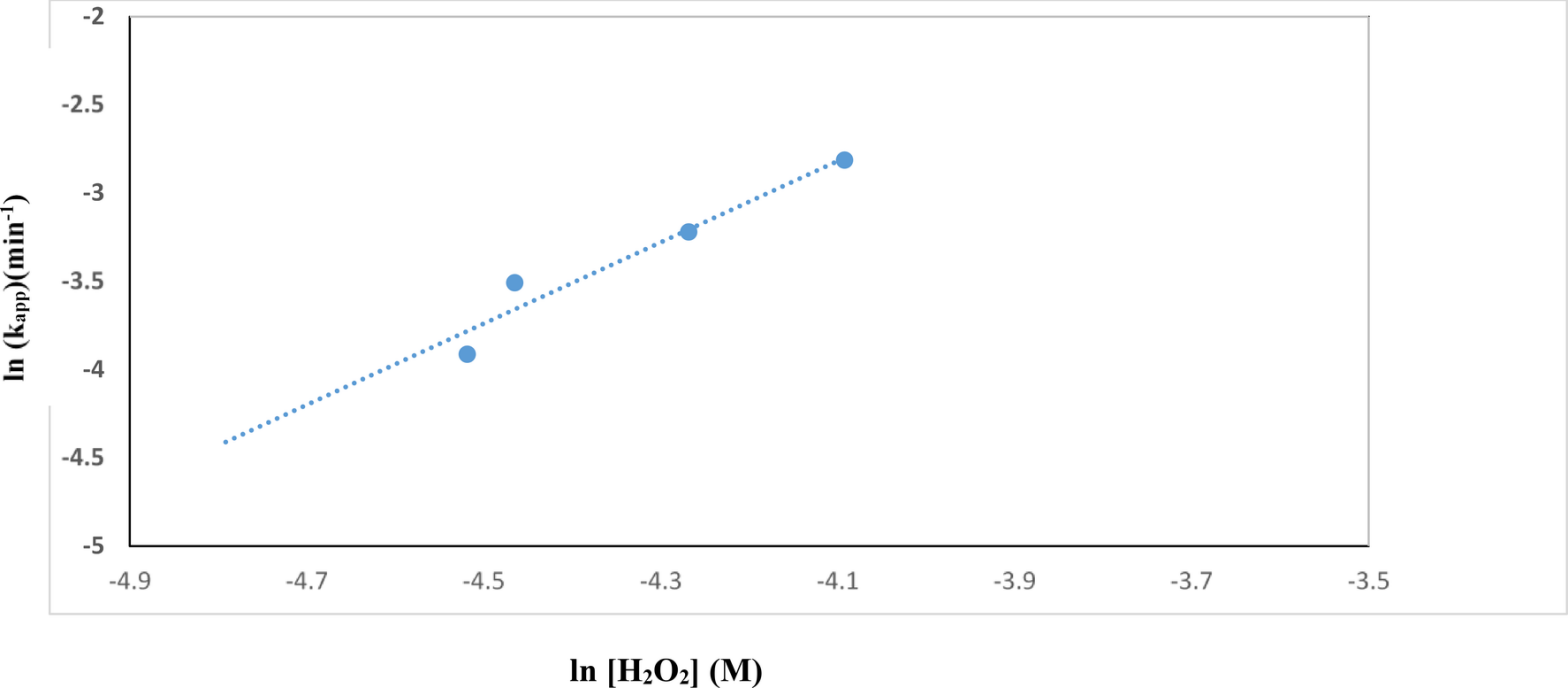
Change of ln k_app_ (min^−1^) with ln [H_2_O_2_] (M) for photocatalytic degradation of acid red 37 dye.

A power law relationship describes the effect of rate-determining species:

3 $${{\text{k}}_{{\text{app}}}}={\text{ K }}{\left[ {{{\text{H}}_{\text{2}}}{{\text{O}}_{\text{2}}}} \right]^{\text{n}}},$$

where k_app_ and K are the apparent and true rate constants, respectively. Plotting the logarithm of k_app_ against the logarithm of the concentration of hydrogen peroxide in Fig. 22 produced a value of 2.3 for the exponent, n, the order of reaction with regard to the oxidant species (Table 1). According to the following reaction, the photogenerated conduction band electrons of TiO_2_–SnO_2_ are more efficiently trapped by H_2_O_2_ than they are by O_2_, which may explain why the rate of dye degradation is increased when using UV/TiO_2_–SnO_2_ (90: 10) wt%/H_2_O_2_ system as compared with UV/TiO_2_–SnO_2_(90:10) wt% alone^22,23^:

4 $${{\text{H}}_{\text{2}}}{{\text{O}}_{\text{2}}}\,+\,{{\text{e}}^ - }_{{({\text{cb}})}}{ \to ^ - }{\text{OH }}{+^ \cdot }{\text{OH}}{\text{.}}$$

Additional oxidizing species (·OH), can aid in the oxidative decay process as a whole, besides the scavenging action which slowing the recombination interaction between photogenerated carriers, (electrons and holes). Since the employed UV source’s main output was at 254 nm, significant photolysis of the H_2_O_2_would have taken place, creating additional hydroxyl radicals^25,26^. Thus,

5 $${{\text{H}}_{\text{2}}}{{\text{O}}_{\text{2}}}\,+\,{\text{hn}} \to {{\text{2}}^ \cdot }{\text{OH}}.$$

By raising the H_2_O_2_ concentrations (2 × 10^−2^–6 × 10^−2^ M), dye oxidation and degradation are accelerated as well as the electron scavenging. Further increasing in H_2_O_2_ concentration to 8 × 10^−2^ M decreases the photocatalytic degradation rate due to the recombination of the resulting of OH radicals which occurs at high concentration of H_2_O_2_^26^.

#### Effect of addition of S2O82− to TiO2–SnO2 (90:10) wt% nanocomposite

The oxygen on the surface of irradiated TiO_2_–SnO_2_ (90: 10) wt% suspension acts as a natural sink for the photogenerated conduction band electrons. The sorbed H_2_O or hydroxyl ions on TiO_2_–SnO_2_surface are subsequently oxidized by the remaining holes to produce the OH radicals. It could be advantageous to use an electron acceptor other than oxygen that is more effective^27–29^.

**Fig. 23:**
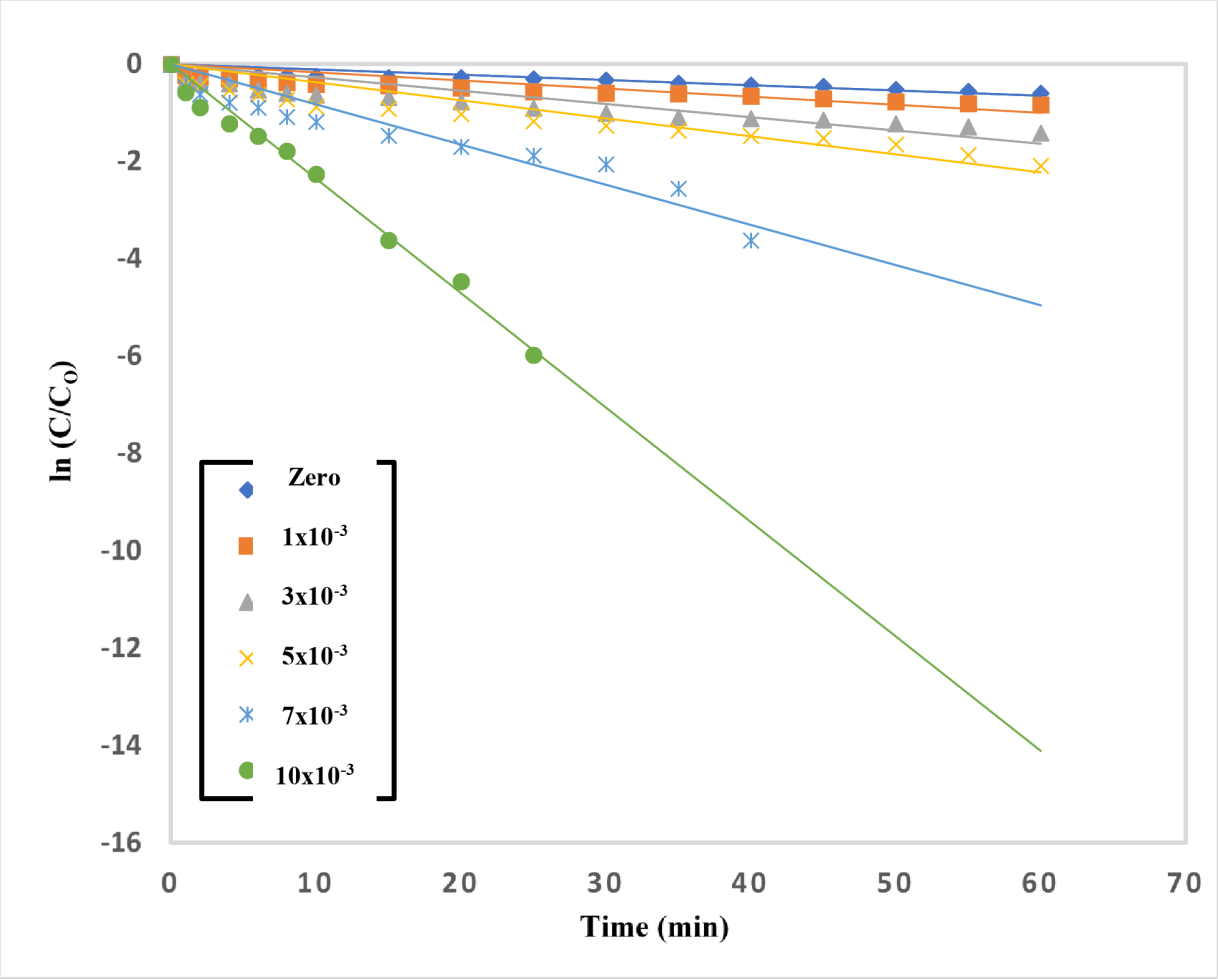
Change of ln (C/C_o_) with time for photocatalytic degradation of acid red 37 dye using 0.4 g/L of TiO_2_–SnO_2_ (90: 10) wt% with different concentrations of S_2_O_8_^2−^ (M).

**Fig. 24:**
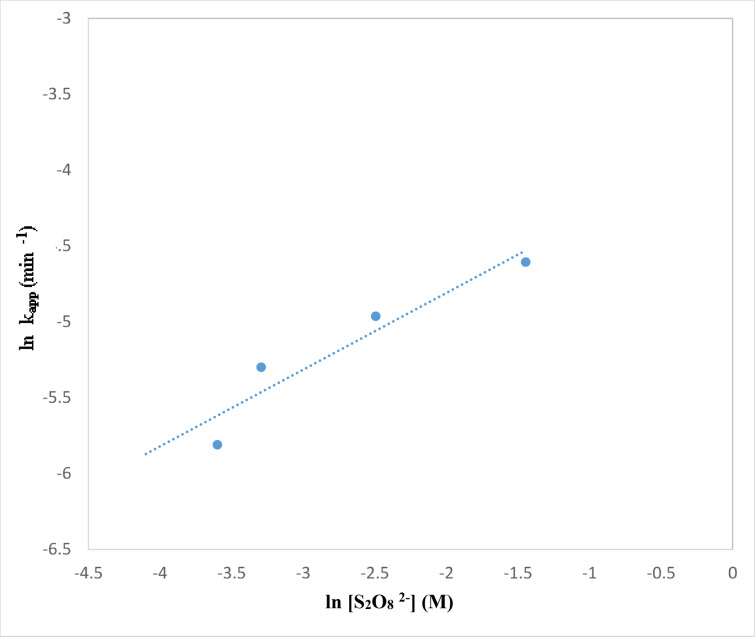
Change of ln k_app_ (min^−1^) with ln [S_2_O_8_^2−^] (M) for photocatalytic degradation of acid red 37 dye.

The impact of persulfate addition to TiO_2_–SnO_2_ (90:10) wt% (0.4 g L^−1^) on the photocatalytic degradation of acid red 37 dye is shown in Fig. 23. The rate of deterioration using TiO_2_–SnO_2_ (90:10) (0.4 g L^−1^) was found to be accelerated by addition of a little amount of persulfate (1.0 × 10^−3^ M). The dye degraded completely and quickly when the persulfate concentration was increased to 10 × 10^−3^ M (2.9 min). Figure 24 shows the reaction rate order with regard to persulfate, and it was determined to be 0.5 (Table 4). The persulfate anions may capture the photogenerated conduction band electrons of TiO_2_–SnO_2_ more than O_2_ or H_2_O_2_ and produce the powerful oxidizer SO_4_^⋅−^^30^.

6 $${{\text{S}}_{\text{2}}}{{\text{O}}_{\text{8}}}^{{{\text{2}} - }}+{\text{ }}{{\text{e}}^ - }_{{({\text{cb}})}} \to {\text{ S}}{{\text{O}}_{\text{4}}}^{{{\text{2}} - }}+{\text{ S}}{{\text{O}}_{\text{4}}}^{{ \cdot - }}$$

Additionally at a wavelength of 254 nm, the sulphate radical anion can interact with the solvent and produces ^·^OH in the following processes^6,31,32^:

7 $${{\text{S}}_{\text{2}}}{{\text{O}}_{\text{8}}}^{{{\text{2}} - }}+{\text{ hn}} \to {\text{ 2 S}}{{\text{O}}_{\text{4}}}^{{ \cdot - }}$$

8 $${\text{S}}{{\text{O}}_{\text{4}}}^{{ \cdot - }}+{\text{ }}{{\text{H}}_{\text{2}}}{\text{O }}{ \to ^ \cdot }{\text{OH}}\,+\,{\text{S}}{{\text{O}}_{\text{4}}}^{{{\text{2}} - }}+{\text{ }}{{\text{H}}^+}$$

As a result, increasing the persulfate concentrations (1 × 10^−3^–10 × 10^−3^ M) improved both the trapping of photogenerated conduction band electrons and the production of SO_4_^·−^ and ^⋅^OH which led to higher rate of photocatalytic degradation.

#### Effect of addition of IO4− to TiO2–SnO2 (90:10) wt% nanocomposite

**Fig. 25:**
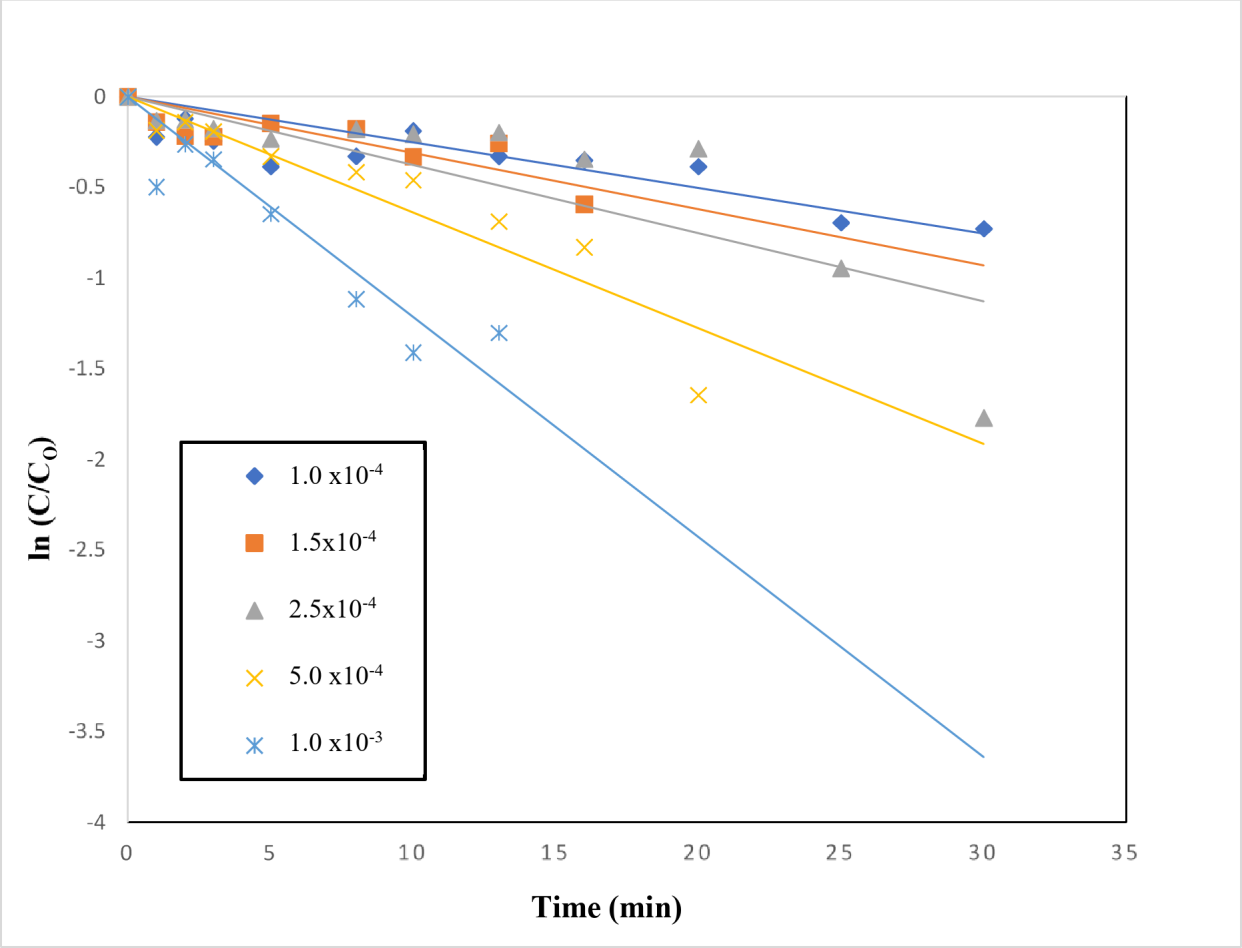
Change of ln (C/C_o_) with time for photocatalytic degradation of acid red 37 dye using 0.4 g/L of TiO_2_–SnO_2_ (90:10) wt% with different concentrations of IO_4_^−^ (M).

The effect of addition of periodate to TiO_2_–SnO_2_ (90:10) wt% (0.4 g L^−1^) for photocatalytic degradation of the studied acid red 37 dye is shown in Fig. 25. It can be seen that the addition of very low concentration of periodate (1 × 10^−4^ M) to UV/TiO_2_–SnO_2_ (90:10) wt% (0.4 g L^−1^) resulted in higher photocatalytic degradation rate compared with UV/TiO_2_–SnO_2_ (90–10) wt% (0.4 g L^−1^) system alone. Also, increasing the periodate concentration to 1 × 10^−3^ resulted in higher photocatalytic degradation of the dye compared with UV/TiO_2_–SnO_2_ (90:10) wt% (0.4 g L^−1^)/H_2_O_2_ or UV/TiO_2_–SnO_2_ (90:10) wt% (0.4 g L^−1^)/S_2_O_8_^2−^ systems, indicating the effectiveness of periodate over peroxide or persulfate.

**Fig. 26:**
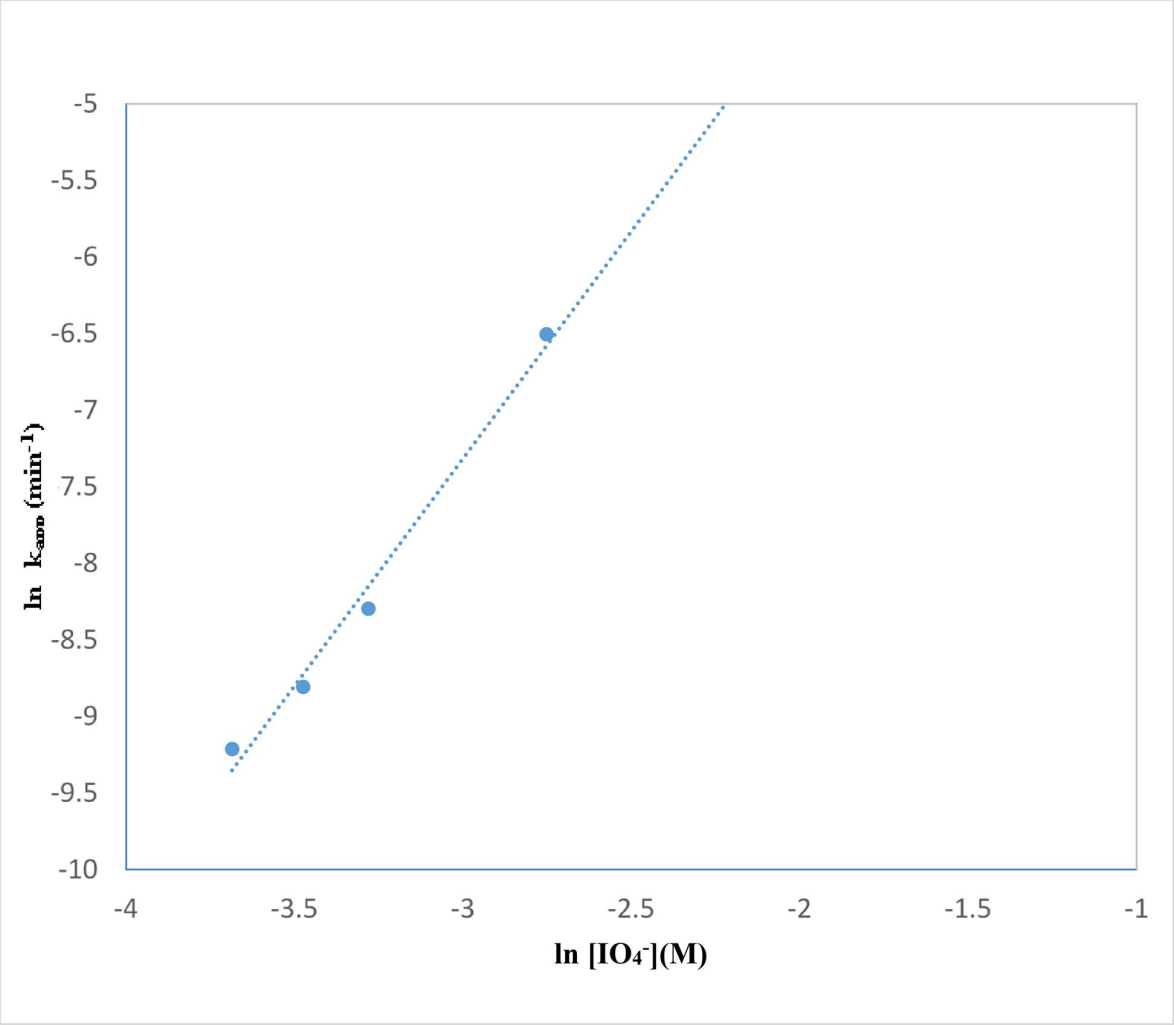
Change of ln k_app_ (min^−1^) with ln [IO_4_^−^] (M) for photocatalytic degradation of acid red 37 dye.

The reaction rate order with respect to periodate was found to be 2.9 (Fig. 26; Table 4). The enhancement of the dye degradation may be due to the scavenging of the photogenerated conduction band electrons of the excited TiO_2_–SnO_2_ by periodate which is more efficient than trapping with O_2_, H_2_O_2_ or S_2_O_8_^2−^as follows^7^:

9 $${\text{I}}{{\text{O}}_{\text{4}}}^{ - }+{\text{8}}{{\text{e}}^ - }_{{({\text{cb}})}}\,+\,{\text{8}}{{\text{H}}^+} \to {\text{ 4}}{{\text{H}}_{\text{2}}}{\text{O}}\,+\,{{\text{I}}^ - }$$

In addition, under UV irradiation (254 nm), a variety of highly reactive radical- and non-radical intermediates (IO_3_⋅, ^⋅^OH, and IO_4_^⋅^) are produced during the photolytic degradation of periodate which enhanced the degradation rate^8^:

10 $${\text{I}}{{\text{O}}_{\text{4}}}^{ - }+{\text{ hn}} \to {\text{ I}}{{\text{O}}_{\text{3}}}^{ \cdot }+{\text{ }}{{\text{O}}^{ \cdot - }},$$

11 $${{\text{O}}^{ \cdot - }}+{\text{ }}{{\text{H}}^+}{ \leftrightarrow ^ \cdot }{\text{OH}}$$

12 $$^{ \cdot }{\text{OH}}\,+\,{\text{I}}{{\text{O}}_{\text{4}}}^{ - } \to {\text{O}}{{\text{H}}^ - }+{\text{I}}{{\text{O}}_{\text{4}}}^{ \cdot }$$

The formation of IO_3_^⋅^, O^⋅−^, ^⋅^OH, and IO_4_^⋅^ as well as the trapping of the photogenerated conduction band electrons of TiO_2_ and SnO_2_ (90:10) wt% are both enhanced by an increase in periodate concentration.

Qamar et al.^33^ have shown that the degradation of chrysoidine R and Acid Red 29 dyes was hastened when peroxide or periodate was added to UV light containing TiO_2_. It was discovered that the response rate followed the sequence:

- UV/TiO_2_/S_2_O_8_^2−^ > UV/TiO_2_/H_2_O_2_ > UV/TiO_2_.

Similarly, Qamar et al.^34^ observed higher rates of chromotrope 2B and amido black 10 B photocatalytic degradation in the presence of H_2_O_2_, KBrO_3_, and (NH_4_)_2_S_2_O_8_. The pace of deterioration was about:

- UV/TiO_2_/BrO_3_^−^ > UV/TiO_2_/S_2_O_8_^2−^ > UV/TiO_2_/H_2_O_2_ > UV/TiO_2_.

Finally, we can showed the total results that for three addition of inorganic antioxidants H_2_O_2_,SO_4_^−^ and IO_4_^−^ in shown Table 4 and compare rate of reaction with TiO_2_ and SnO_2_ alone then after addition inorganic antioxidant.

**Table 4:** Collective data of apparent rate constants, half life times and reaction orders for photocatalytic degradation of acid red 37 dye.

Catalytic system	Concentration	k_app_ (min^−1^)	t_1/2_ (min)	Apparent reaction order (*n*)
UV/SnO_2_ (g/L)	0.4	2 × 10^−4^	3460	–
UV/TiO_2_ (g/L)		28 × 10^−4^	247.1	
UV/TiO_2_–SnO_2_ (97:3) (g/L)		38 × 10^−4^	182.1	
UV/TiO_2_–SnO_2_ (90:10) (g/L)		72 × 10^−4^	96.1	
UV/TiO_2_–SnO_2_ (80:20) (g/L)		35 × 10^−4^	197.7	
UV/TiO_2_–SnO_2_ (90:10) wt% (0.4 g/L)/H_2_O_2_ (M)	2 × 10^−2^	789 × 10^−4^	8.7	2.3
	3 × 10^−2^	883 × 10^−4^	7.8	
	4 × 10^−2^	943 × 10^−4^	7.3	
	6 × 10^−2^	997 × 10^−4^	6.9	
	8 × 10^−2^	698 × 10^−4^	9.9	
UV/TiO_2_–SnO_2_ (90:10) wt% (0.4 g/L)/S_2_O_8_^2−^ (M)	1 × 10^−3^	166 × 10^−4^	41.7	0.5
	3 × 10^−3^	273 × 10^−4^	25.3	
	5 × 10^−3^	371 × 10^−4^	18.6	
	7 × 10^−3^	826 × 10^−4^	8.3	
	10 × 10^−3^	2352 × 10^−4^	2.9	
UV/TiO_2_–SnO_2_ (90:10) wt% (0.4 g/L)/IO_4_^−^ (M)	1.0 × 10^−4^	251 × 10^−4^	27.6	2.9
	1.5 × 10^−4^	310 × 10^−4^	22.3	
	2.5 × 10^−4^	376 × 10^−4^	18.4	
	5.0 × 10^−4^	638 × 10^−4^	10.8	
	1 × 10^−3^	1214 × 10^−4^	5.7	

## Materials and methods

### Artificial polluted wastewater preparation

An aqueous solution of Acid Red 37 dye (1.04 × 10^–4^ M) were prepared in double distilled water as a model for wastewater pollutant. The pH of TiO_2_–SnO_2_/dye suspension was 6.1. Addition of H_2_O_2_, NaIO_4_ or Na_2_S_2_O_**8**_ to TiO_2_–SnO_2_/dye suspension decreases the pH below 6.1. A few drops of an alkali were added to adjust the pH to its original value 6.1.

### Preparation of TiO2–SnO2 nanocomposites

TiO_2_–SnO_2_ nanocomposites were prepared through a solid-state reaction route. The starting material was TiO_2_ (99.58%). 3, 7, 10, 15 and 20 wt% of SnO_2_ powder have been added to TiO_2_. The powders have been mixed uniformly by grinding in mortar with pestle for 3 h to get fine powders. The resultant powders have been annealed in air at 500 °C for 3 h in an electric muffle furnace^35^.

### Schematic diagram of batch photoreactor

**Fig. 27:**
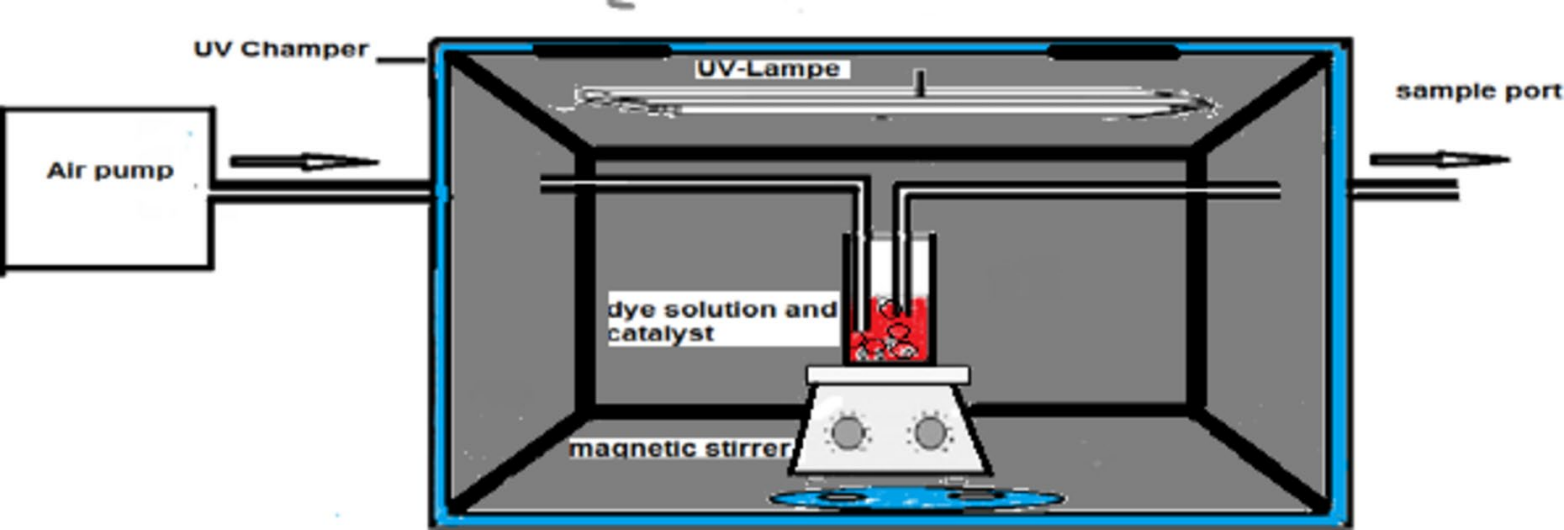
Schematic diagram of the batch photoreactor.

The reactor used for studying the photodegradation of the dye in all experiments was a batch photoreactor as shown Fig. 27. To increase solution fluidization and access oxygen for Eq. (13)

13 $${\text{Adsorbed oxygen}}:{\text{ }}\left( {{{\text{O}}_{\text{2}}}} \right){\text{ads}}\,+\,{\text{e}} - \to {{\text{O}}_{\text{2}}}^{{ - \cdot }},$$

air was blown into the reaction solution using an air pump at a flow rate of 10 m^3^/h. Blowing cooled air into the solution eliminated the lamp’s heat effect and kept the temperature at around 25 °C^36^. It is consists of a glass container (250 ml). The contents (TiO_2_/dye, SnO_2_/dye, TiO_2_–SnO_2_/dye, or TiO_2_–SnO_2_/dye/oxidant) of the container were agitated by a magnetic stirrer and kept purged with air at a rate of 3000 ml min^−1^. The dye solution was stirred with TiO_2_–SnO_2_ nanocomposite in the dark for 30 min before irradiation with UV to obtain equilibrium adsorption. Irradiation was carried out with a tubular low pressure mercury lamp (total rating 43 W, total UV output at 513 nm 13.4 W, and length 120 cm, Voltarc Tubes Inc., USA) was located 10 cm from the surface of the dye solution. The total intensity reaching the slurry solution was measured using a UVX radiometer (UV Products Ltd., Cambridge) equipped with a sensor with peak sensitivity at 254 nm was 4 mW cm^−2^. Samples of dye solution (1.0 × 10^−4^ mol/L) which prepared in distilled water as a model for wastewater pollutants were removed from the container via a sample port periodically and measured after filtration through 0.2 μm polyether sulfone membrane. All photodegradation experiments were done in a temperature of 22 ± 2 °C.

### Measurement of photocatalytic activity

The photocatalytic performance of native TiO_2_, SnO_2_ nanoparticles and TiO_2_–SnO_2_nanocomposites was investigated by the decomposition of acid red 37 dye in an aqueous solution under UV light (254 nm). Absorbance was readily described by Beer-Lambert law^37^

14 $${\text{A}}\,=\,\varepsilon {\text{LC}}$$

Where A is absorbance, ε is the molar extinction coefficient, L is the light path length (1.00 cm) and C is the concentration. The absorbance which given at each irradiation time was related to a specific dye concentration using a calibration curve for acid red 37 dye solutions with different concentrations. The percent of degradation of the dye could be calculated according to equation (15) :

15 $${\text{Degradation }}\% {\text{ }}={\text{ }}{{\text{C}}_{\text{o}}} - {\text{ C}}/{{\text{C}}_{\text{o}}} \times {\text{ 1}}00,$$

Where C_o_ = initial concentration of dye solution, C = concentration of dye solution after photoirradiation at time t.

## Conclusions

The photocatalytic degradation of the investigated acid red 37 dye using TiO_2_ nanoparticles was found to be higher than that of SnO_2_. The photocatalytic activity results showed that the couple oxide TiO_2_–SnO_2_ exhibited much higher photocatalytic activity than the native SnO_2_ and native TiO_2_.Heterogeneous photocatalytic degradation of the investigated acid red 37 dye using TiO_2_–SnO_2_ nanocomposite with different wt% (3–20) indicated that: UV/TiO_2_–SnO_2_ (90:10) wt% performing the higher photocatalytic degradation of the dye than other ratios. Heterogeneous photocatalytic degradation using UV/TiO_2_–SnO_2_ (90:10)/Oxidant (H_2_O_2_, S_2_O_8_^2−^, or IO_4_^−^) systems was found to be more effective than UV/SnO_2_ or UV/TiO_2_ and follows the following order:

- UV/SnO_2_ < UV/TiO_2_ < TiO_2_–SnO_2_ (90:10) < UV/TiO_2_–SnO_2_ (90:10)/H_2_O_2_ < UV/TiO_2_–SnO_2_ (90:10)/Na_2_S_2_O_8_ < UV/TiO_2_–SnO_2_ (90:10)/NaIO_4_.

## Data Availability

All data generated or analyzed during this study are included in this article.

## References

[1] Kadem, A. J., Tan, Z. M., Suntharam, N. M., Pung, S. Y. & Ramakrishnan, S. Synthesis of CuO, ZnO and SnO_2_ coupled TiO_2_ photocatalyst particles for enhanced photodegradation of rhodamine B dye. *Bull. Chem. React. Eng. Catal.* **18**(3), 506–520 (2023).

[2] .Raub, A. A. M. et al. Photocatalytic activity enhancement of nanostructured metal-oxides photocatalyst: a review. *Nanotechnology* **1**, 1 (2024). 10.1088/1361-6528/ad33e8 38484390

[3] Fatimah, I. et al. Ultrasound-assisted phyto-mediated synthesis of SnO_2_ nanoparticles as photocatalyst in tetracycline photocatalytic oxidation. *Inorg. Chem. Commun.* **1**, 112096 (2024).

[4] Yang, L. R. et al. Functionalizing slag woolfibers with photocatalytic activity by anatase TiO_2_ and CTAB modification. *Ceram. Int.* **44**, 5842–5847 (2018).

[5] Gandolfo, A. et al. Unexpectedly high levels of organic compounds released by indoor photocatalytic paints. *Environ. Sci. Technol.* **52**, 328–11337 (2018). 10.1021/acs.est.8b03865 30188114

[6] Panwar, S., Upadhyay, G. K. & Purohit, L. P. Gd-doped ZnO: TiO_2_ heterogenous nanocomposites for advance oxidation process. *Mater. Res. Bull.* **145**, 111534 (2022).

[7] Obotey Ezugbe, E. & Rathilal, S. Membrane technologies in wastewater treatment: a review. *Membranes* **10**(5), 89 (2020). 32365810 10.3390/membranes10050089 PMC7281250

[8] Fernandes, A., Makoś, P., Wang, Z. & Boczkaj, G. Synergistic effect of TiO_2_ photocatalytic advanced oxidation processes in the treatment of refinery effluents. *Chem. Eng. J.* **391**, 123488 (2020).

[9] Kumar, P. S. S., Raj, M. R. & Anandan, S. Nanoporous Au–TiMCM-41—An inorganic hybrid photocatalyst toward visible photooxidation of methyl orange. *Sol. Energy Mater. Sol. Cells* **94**(10), 1783–1789 (2010).

[10] Vinu, R., Akki, S. U. & Madras, G. Investigation of dye functional group on the photocatalytic degradation of dyes by nano-TiO_2_. *J. Hazard. Mater.* **176**(1–3), 765–773 (2010). 20018445 10.1016/j.jhazmat.2009.11.101

[11] Sadik, W. A., El-Demerdash, A. G. M., Nashed, A. W., Mostafa, A. A. & Hamad, H. A. Highly efficient photocatalytic performance of Cu_2_O@ TiO_2_ nanocomposite: influence of various inorganic oxidants and inorganic anions. *J. Mater. Res. Technol.* **8**(6), 5405–5414 (2019).

[12] Wang, H. et al. Engineering of SnO_2_/TiO_2_ heterojunction compact interface with efficient charge transfer pathway for photocatalytic hydrogen evolution. *Chin. Chem. Lett.* **34**(1), 107125 (2023).

[13] Thanh, N. T., Maclean, N. & Mahiddine, S. Mechanisms of nucleation and growth of nanoparticles in solution. *Chem. Rev.* **114**(15), 7610–7630 (2014). 25003956 10.1021/cr400544s

[14] Kaur, N. & Singh, V. Current status and future challenges in ionic liquids, functionalized ionic liquids and deep eutectic solvent-mediated synthesis of nanostructured TiO_2_: a review. *N. J. Chem.* **41**, 2844–2868 (2017).

[15] Wang, Z. et al. Rapid preparation of terbium-doped titanium dioxide nanoparticles and their enhanced photocatalytic performance. *R. Soc. Open. Sci.* **6**(10), 191077 (2019). 31824714 10.1098/rsos.191077 PMC6837207

[16] Tangale, N. P. et al. Synthesis of Sn-containing anatase (TiO_2_) by sol-gel method and their performance in catalytic water splitting under visible light as a function of tin content. *Mater. Lett.* **171**, 50–54 (2016).

[17] Molaei, M. J. Principles, mechanism, and identification of S-scheme heterojunction for photocatalysis: a critical review. *J. Am. Ceram. Soc.* **1**, 1 (2024).

[18] Suriya, P., Prabhu, M., Ezhilselvi, V. & Jagannathan, K. Improvement of power conversion efficiency by tailoring of energy band gaps in Ag doped TiO_2_–SnO_2_ nanocomposites. *Phys. B Condens. Matter* **670**, 415359 (2023).

[19] Saleh, T. A., Mustaqeem, M. & Khaled, M. Water treatment technologies in removing heavy metal ions from wastewater: a review. *Environ. Nanatechnol. Monit. Manag.* **17**, 100617 (2022).

[20] Mannaa, M. A., Hassan, S. M. & Ahmed, A. I. Enhancement the photocatalytic activity of the SnO_2_/TiO_2_ nanocrystals under UV-visible light. *Int. J. Mod. Chem.* **9**(1), 84–92 (2017).

[21] Rajput, R. B., Jamble, S. N. & Kale, R. B. A review on TiO_2_/SnO_2_ heterostructures as a photocatalyst for the degradation of dyes and organic pollutants. *J. Environ. Manag.* **307**, 114533 (2022). 10.1016/j.jenvman.2022.114533 35121365

[22] Mousa, S. A., Abdallah, H., Ibrahim, S. S. & Khairy, S. A. Enhanced photocatalytic properties of graphene oxide/polyvinylchloride membranes by incorporation with green prepared SnO_2_ and TiO_2_ nanocomposite for water treatment. *Appl. Phys. A* **129**(12), 831 (2023).

[23] Hussein, F. M., Alani, R. R. & AL-MOKARAM, A. L. I. Synthesis and photocatalytic activity of TiO_2_-coupled SnO_2_ nanoparticles prepared by sol–gel technique. *Egypt. J. Chem.* **65**(10), 551–559 (2022).

[24] Enesca, A. Enhancing the photocatalytic activity of SnO_2_-TiO_2_ and ZnO-TiO_2_ tandem structures toward indoor air decontamination. *Front. Chem.* **8**, 583270 (2020). 33324610 10.3389/fchem.2020.583270 PMC7723902

[25] Messaadi, C. et al. Synthesis and characterization of SnO_2_-TiO_2_ nanocomposites photocatalysts. *Curr. Nanosci.* **15**(4), 398–406 (2019).

[26] Negishia, N. et al. Photocatalytic detoxification of aqueous organophosphorus by TiO_2_ immobilized silicagel. *Appl. Catal. B* **128**, 105–118 (2012).

[27] Zhao, X., Liu, X., Yu, M. M., Wang, C. & Li, J. The highly efficient and stable Cu, Co, Zn-porphyrinTiO_2_ photocatalysts with heterojunction by using fashioned one-step method. *Dyes Pigm.* **138**, 648–656 (2017).

[28] Gomeza, S., Marchenaa, C. L., Pizziob, L. & Pierella, L. Preparation and characterization of TiO_2_/HZSM-11 zeolite for photodegradation of dichlorvos in aqueous solution. *J. Hazard. Mater.* **258–259**, 19–26 (2013). 10.1016/j.jhazmat.2013.04.030 23692679

[29] Kuo, C. S., Lin, C. F. & Hong, P. K. Photocatalytic degradation of methamphetamine by UV/TiO_2_—kinetics, intermediates, and products. *Water Res.* **74**, 1–9 (2015). 25703658 10.1016/j.watres.2015.01.043

[30] Putluru, S. R. & Schill, L. H. Mn/TiO_2_ and Mn-Fe/TiO_2_ catalysts synthesized by deposition precipitation—promising for selective catalytic reduction of NO with NH_3_ at low temperatures. *Appl. Catal. B* **165**, 628–635 (2015).

[31] Nam, Y., Li, L., Lee, J. Y. & Prezhdo, O. V. Size and shape effects on charge dynamics of TiO_2_ nanoclusters. *J. Phys. Chem.* **C122**, 5201–5208 (2018).

[32] Kaur, N. & Singh, V. Current status and future challenges in ionic liquids, functionalized ionic liquids and deep eutectic solvent-mediated synthesis of nanostructured TiO_2_: a review. *N. J. Chem.* **41**, 2844–2868 (2017).

[33] Alosfur, F. K. M., Ridha, N. J., Jumali, M. H. H. & Radiman, S. One-step formation of TiO_2_ hollow spheres via a facile microwave-assisted process for photocatalytic activity. *Nanotechnology* **29**(14), 145707 (2018). 29384494 10.1088/1361-6528/aaabee

[34] Verma, A., Prakash, N. T. & Toor, A. P. An efficient TiO_2_ coated immobilized system for the degradation studies of herbicide isoproturon: Durability studies. *Chemosphere* **109**, 7–13 (2014). 10.1016/j.chemosphere.2014.02.051 24873700

[35] Hamad, H. et al. The superior photocatalytic performance and DFT insights of S-scheme CuO@ TiO_2_ heterojunction composites for simultaneous degradation of organics. *Sci. Rep.* **12**(1), 2217 (2022). 35140284 10.1038/s41598-022-05981-7 PMC8828870

[36] Abdollahi, Y., Abdullah, A. H., Zainal, Z. & Yusof, N. A. Photodegradation of m-cresol by zinc oxide under visible-light irradiation. *Int. J. Chem.* **3**(3), 31 (2011).

[37] Gomez, S., Marchena, C. L., Pizzio, L. & Pierella, L. Preparation and characterization of TiO_2_/HZSM-11 zeolite for photodegradation of dichlorvos in aqueous solution. *J. Hazard. Mater.* **258**, 19–26 (2013). 23692679 10.1016/j.jhazmat.2013.04.030

